# Advances in Materials, Sensors, and Integrated Systems for Monitoring Eye Movements

**DOI:** 10.3390/bios12111039

**Published:** 2022-11-17

**Authors:** Seunghyeb Ban, Yoon Jae Lee, Ka Ram Kim, Jong-Hoon Kim, Woon-Hong Yeo

**Affiliations:** 1 School of Engineering and Computer Science, Washington State University, Vancouver, WA 98686, USA; 2 IEN Center for Human-Centric Interfaces and Engineering, Institute for Electronics and Nanotechnology, Georgia Institute of Technology, Atlanta, GA 30332, USA; 3 School of Electrical and Computer Engineering, Georgia Institute of Technology, Atlanta, GA 30332, USA; 4 George W. Woodruff School of Mechanical Engineering, Georgia Institute of Technology, Atlanta, GA 30332, USA; 5 Department of Mechanical Engineering, University of Washington, Seattle, WA 98195, USA; 6 Wallace H. Coulter Department of Biomedical Engineering, Georgia Tech and Emory University School of Medicine, Atlanta, GA 30332, USA; 7 Neural Engineering Center, Institute for Materials, Institute for Robotics and Intelligent Machines, Georgia Institute of Technology, Atlanta, GA 30332, USA

**Keywords:** eye movement monitoring, human–machine interface, wearable technology, biopotential monitoring, electrooculography

## Abstract

Eye movements show primary responses that reflect humans’ voluntary intention and conscious selection. Because visual perception is one of the fundamental sensory interactions in the brain, eye movements contain critical information regarding physical/psychological health, perception, intention, and preference. With the advancement of wearable device technologies, the performance of monitoring eye tracking has been significantly improved. It also has led to myriad applications for assisting and augmenting human activities. Among them, electrooculograms, measured by skin-mounted electrodes, have been widely used to track eye motions accurately. In addition, eye trackers that detect reflected optical signals offer alternative ways without using wearable sensors. This paper outlines a systematic summary of the latest research on various materials, sensors, and integrated systems for monitoring eye movements and enabling human-machine interfaces. Specifically, we summarize recent developments in soft materials, biocompatible materials, manufacturing methods, sensor functions, systems’ performances, and their applications in eye tracking. Finally, we discuss the remaining challenges and suggest research directions for future studies.

## 1. Introduction

### 1.1. Recent Advances in Eye Movement Monitoring

**Figure 1:**
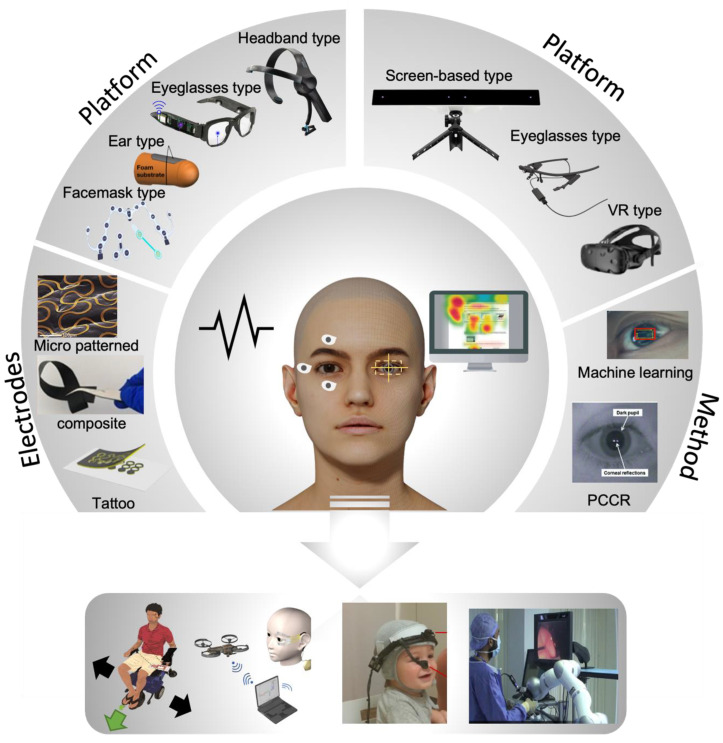
Recent advances in eye tracking sensors, systems, and methods (Screen-based type, Eyeglasses type (right), and VR type: Reprinted under terms of the CC-BY license [3]. Copyright 2020, the authors. Published by MDPI), (Headband type: Reprinted with permission [4]. Copyright 2019 Elsevier), (Eyeglasses type (left): Reprinted with permission [5]. Copyright 2020 American Chemical Society), (Ear type: Reprinted under terms of the CC-BY license [6]. Copyright 2017, the Authors. Published by Springer Nature), (Facemask type: Reprinted with permission [7]. Copyright 2019 Elsevier), (Metal membrane: Reprinted with permission [8]. Copyright 2013 John Wiley and Sons), (Composite: Reprinted with permission [9]. Copyright 2021 American Chemical Society), (Tattoo: Reprinted with permission [10]. Copyright 2017 American Chemical Society), (Smart wheelchair: Reprinted with permission [1]. Copyright 2017, Elsevier B.V), (Drone control: Reprinted under terms of the CC-BY license [11]. Copyright 2018, the Authors. Published by Springer Nature), (Infant Analysis: Reprinted with permission [12]. Copyright 2020 Elsevier), (laparoscopic surgery: Reprinted under terms of the CC-BY license [13]. Copyright 1969, the authors. Published by Elsevier Ltd.), (PCCR: Reprinted with permission [14]. Copyright 2017 Springer Nature), (Machine learning: Reprinted under terms of the CC-BY license [15]. Copyright 2021, the Authors. Published by MDPI).

Electrophysiology signals are often used for health status indicators related to all human activities and various applications. Recent advances in wearable technologies and video monitoring systems for eye movement enabled various types of human–machine interface (HMI) [1,2]. Among them, electrooculograms (EOGs), measured by surface-mounted electrodes, have been widely used to track eye movements. Existing devices for EOG signal measurements cause discomfort due to their bulky and rigid properties. Moreover, the conventional EOG measurement device can only be performed in a stationary lab setup. Recent advances in wearable technologies, such as soft materials, manufacturing technology, and electronic chip packaging, are improved to compensate for existing problems, and these advances directly interact with electronic, mechanical, or computing elements, a collective practice known as HMI. Moreover, recent enhancements in computing power have made it possible for real-time eye tracking to monitor changes in eye motions with different types of cameras. Eye tracking is deployed in various research areas, including psychology, neuroscience, and marketing, to understand human intentions and responses (Figure 1).

### 1.2. Electrooculogram-Based Approaches for Human–Machine Interfaces

EOG is one of the technologies for tracking eye movements by measuring the potential via the positively charged cornea and negatively charged retina [1]. The measured signal results are called EOG. Generally, the range of the measured EOG signals is from 50 μV to 3500 μV depending on the amount of light incident on the retina [16,17]. It is common practice to use generic electrical sensors for EOG detection. Since these conventional EOG devices have rigid properties, wearable device platforms based on soft electronics and wireless data communications could offer an improved user experience. The concept of a wearable EOG device includes measuring the EOG signal in a wearable environment for providing smart diagnostics and application controllers with embedded signal processing such as machine learning algorithms. Building a wearable EOG system requires electrodes, platforms, and signal processing to analyze the EOG signal. Electrodes are essential for measuring bio-potentials. Existing metal-based electrodes are flat in shape with gels for adhesion. Flat-shaped electrodes are not suitable for human skin due to skin deformations. The gel also causes several skin issues such as skin irritation and poor breathability. Due to the above problems, research groups recently studied electrodes with flexible form factors, biocompatible materials, and cost-effective processes. As an example, polymer-based electrodes (sponge [18,19,20], textile [21], and hydrogel [22,23,24,25,26,27,28,29]) have been utilized because of their advantages such as good mechanical flexibility, low density, the ease of processing, and low costs. Recent advances in microfabrication and print technologies enabled new ways to design micro-patterned electrodes (gold [1,30] and graphene [11]). Due to the development of these technologies and 3D printing, designing wearable platforms has become possible, such as eyeglass types [5,31,32,33,34,35,36,37,38,39], face mask types [7,40,41,42], ear plug types [43,44,45,46,47], and headband types [48,49,50,51,52,53,54,55,56,57,58] for various applications. Previously, various controllers for an HMI such as wheelchairs [1,4,51,52], drones [11,59], game interfaces [5,36,47,60,61], and virtual keyboards [34,38,51,62] were created by using only an EOG signal. Recently, various healthcare monitoring systems [7,40,41,44,45,63] and medical health status analyses [64,65,66] have been conducted using both the electroencephalogram (EEG) and EOG with signal processing, such as machine learning algorithms [1,5,36,52]. The studies mentioned above show that the advancement in wearable EOG devices makes it easier to use HMI in daily life.

### 1.3. Screen-Based Eye Tracking Technology

Over the past few decades, screen-based eye trackers have been successfully used for several applications to find out the involuntary or voluntary recognition of human intention by tracking the gaze point on the screen. The intuitive human intention could be delivered to the human–machine interface with the exact coordinates of the gaze point on an object or screen. The eye tracker-based signal computation process could be represented by two types of methods: machine learning [67,68,69,70] and pupil center-corneal reflection (PCCR) [71,72,73]. Each technique required several cameras to create a trace map or to detect gaze points on the screen. These fundamentals of eye movement and eye gaze analysis are the basic parameters of heat maps, including the area of interest, time to first fixation, dwell time, and integration model. Moreover, recent developments in real-time computer devices led to the emergence of mobile and stationary eye tracker platforms to change daily lives. A new advancement in optical device-based mobile eye tracking systems presents comprehensive nonintrusive human gaze points [73,74]. The form factors of recent eye tracking devices are eyeglasses, screen-attached cameras, and screen-mounted goggles. These non-invasive eye tracking platforms allow the collection of comprehensive eye information data. Various applications have attempted to analyze human attention and intention from algorithmic reproduction using eye tracking data. Here, we focus on an all-inclusive review of eye tracking methods (such as EOG and video monitoring) and wearable systems, including electrodes, platforms, and signal-processing technologies for various applications (Figure 1). We summarize the types of platforms and the characteristics of the electrodes, including biocompatible, mechanical, and electrical properties. In addition, the signal processing strategy is discussed in view of targeted applications and data sets. Moreover, we summarize the principle, platform, and applications of eye tracking employed throughout many fields of psychology, medical examination, cognitive science, and disease diagnosis. Finally, we discuss future works related to next-generation eye tracking technologies, promoting continuous development via cooperation with various technologies.

## 2. EOG Signals

**Figure 2:**
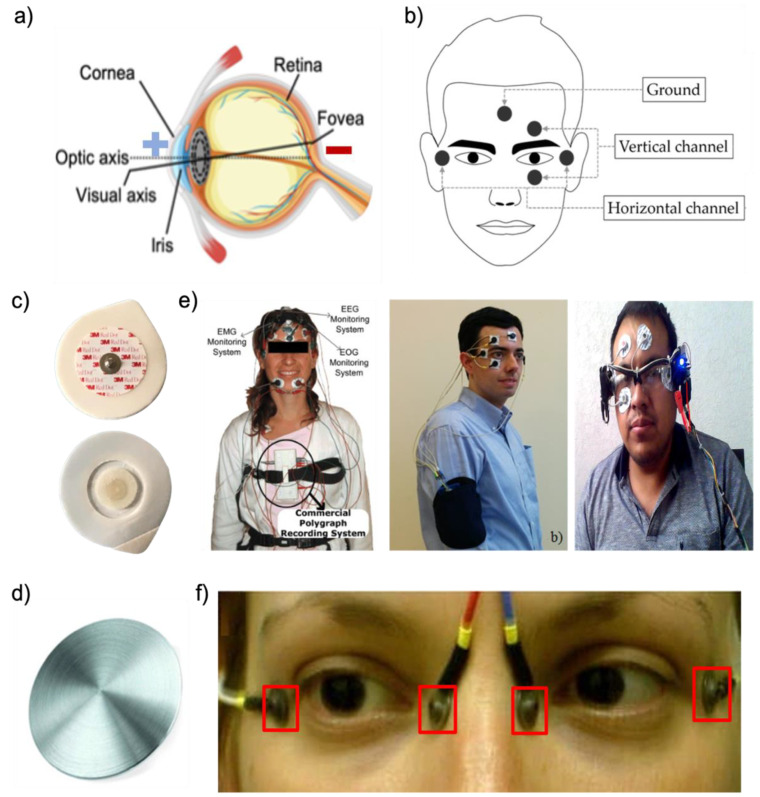
EOG detection systems. (**a**) An anatomical illustration of the eye (cornea being positive and retina being negative). (**b**) Positions of electrodes for EOG detection (reprinted under terms of the CC-BY license [51]. Copyright 2017, the authors. Published by MDPI). (**c**) Conventional Ag/AgCl electrodes. (**d**) Example of a stiff material (metal disc) (Reprinted with permission [86]. Copyright 2013 Institute of Physics and Engineering in Medicine). (**e**) Exiting EOG devices with conventional gel electrodes (Left: Reprinted with permission [81]. Copyright 2009 Elsevier, Middle: reprinted with permission [60]. Copyright 2018 Elsevier, Right: reprinted under terms of the CC-BY license [37]. Copyright 2021, the Authors. Published by MDPI). (**f**) Exiting EOG devices with dry metal electrodes (Reprinted under terms of the CC-BY-NC license [87]. Copyright 2018, the Authors. Published by Springer Nature).

A key metric of the positive potential on the cornea and negative potential on the retina is shown in Figure 2a. The EOG signal is acquired from electrodes around the eyes or forehead, as described for various EOG platforms in the above section. Because electrodes can transduce bioelectric activities within the body into electrical currents, electrodes are essential components for obtaining EOG signals. For EOG collection, electrodes will be positioned on the user’s face, as shown in Figure 2b. Two electrodes are placed next to the lateral canthus of each eye to detect horizontal (i.e., left and right) eye movements. To detect vertical (i.e., up and down) eye movements, two electrodes are placed on the top and bottom of one of the eyes. An electrode is placed in the middle of the forehead, chin, or back of the ear as a reference. The electrode placed at the lateral canthus of the left eye is connected to the positive terminal of the horizontal channel, and another electrode placed at the lateral canthus of the right eye is connected to the negative terminal of the vertical channel. Other electrode sets for vertical channels are similar to those for horizontal channels. One electrode placed at the top of the left eye is connected to the positive terminal of the vertical channel, and another electrode placed at the bottom of the left eye is connected to the negative terminal of the vertical channel. According to the positions of the electrodes mentioned above, we explain how EOG signals are measured. When the electrodes capture the eye movements, the electrode nearby the eye’s direction detects the positive potential from the cornea, and another electrode opposite of the eye’s direction detects the negative potential from the retina. For example, when the eyes move to the right, the pair of horizontal electrodes detect the negative potential. Alternatively, when the eyes move to the left, the pair of horizontal electrodes set detects the positive potential. Similarly to the horizontal electrodes above, the vertical electrodes measure the potential according to the direction of the eye. When the eyes look up, vertical electrodes detect the positive potential, and when they look down, vertical electrodes detect the negative potential. The blink signal is not an EOG signal. The EOG signal is the electric potential difference between the retina and the cornea. However, the blink signal is an electromyography (EMG) signal from the movement of the eye muscle. EMG measures electrical muscle responses in response to stimulation in the nerves. EOG waveforms show the peaks when the eyes move left, right, up, and down from the first position. Here, this section introduces various types of electrodes, such as hydrogel, fiber, polymer, and micro-patterned types, which can solve problems with existing gel and dry electrodes.

### 2.1. Existing Electrodes

Wearable EOG devices that require electrodes and wearable platforms can measure changes in eye movements during daily activities [1,75]. Conventional electrodes, wet or dry silver/silver chloride (Ag/AgCl), are generally used to measure EOG signals [76,77,78,79,80]. For example, wet Ag/AgCl electrodes are used for the analysis of various activity recognition fields [65,81,82] or HMI controllers [37,60,62,83]. Dry flat Ag/AgCl electrodes are used on various wearable platforms such as eyeglasses [35], head caps [55], and goggles [34]. From the perspective of wet Ag/AgCl electrodes (Figure 2c), conductive gel dehydration results in electrode performance degradation over time. The conductive gel can cause pain and skin rashes when it is used on human skin [1] and might cause a short circuit if electrodes are placed close to each other [84]. Poor breathability is also one of the gel electrode’s drawbacks. It is hard to use for wearable platforms such as eyeglasses because the gel electrodes are too bulky for mounting around the nose. On the other hand, dry Ag/AgCl electrodes are better for the long-term measurements of the EOG signal than wet electrodes (Figure 2d). However, dry electrodes are thick and stiff, leading to a high electrode–skin impedance and vulnerability to motion artifacts with poor contact on the delicate skin around the eyes [85]. The wearable EOG device systems with existing electrodes, such as conventional wet or dry electrodes, are often bulky and complex, as shown in Figure 2e,f. Therefore, many research groups introduced non-invasive, bio-compatible, and high-quality recording electrode types to address the above issues. This section introduces the various types of electrodes, such as hydrogel, fiber, polymer, and micro-patterned types (Table 1).

#### 2.1.1. Composite Electrodes

**Figure 3:**
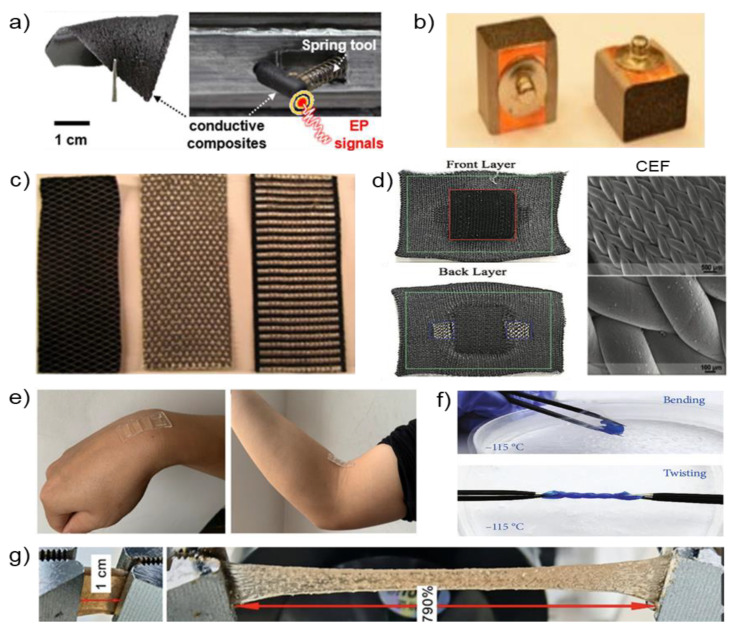
Examples of composite electrodes. (**a**) Carbon nanotubes embedded in a printed eyeglass (Reprinted with permission [5]. Copyright 2020 American Chemical Society). (**b**) Conductive polymer foam based on urethane and taffeta materials (Reprinted with permission under the terms of the CC-BY license [38]. Copyright 2021, the Authors. Published by MDPI). (**c**) Silver embroidered electrode and electrode-lead connection (Reprinted with permission under the terms of the CC-BY license [21]. Copyright 2021, the Authors. Published by MDPI). (**d**) 3D-Knit dry electrodes using conductive elastomeric fibers with CEF (Reprinted with permission [90]. Copyright 2022 Wiley-VCH GmbH). (**e**) Photographs demonstrating adhesion of the flexible hydrogel (Reprinted with permission under the terms of the CC-BY license [97]. Copyright 2021, the authors. Published by MDPI). (**f**) Tortuosity of the proposed hydrogel at −115 °C (Reprinted with permission under the terms of the CC-BY license [98]. Copyright 2021, the Authors. Published by IOP). (**g**) Photographs demonstrating the stretchability of the starch hydrogel (Reprinted with permission [61]. Copyright 2022 Wiley-VCH GmbH).

The composite electrodes are introduced to compensate for the drawbacks of conventional electrodes, such as skin irritation or motion artifacts from human skin. Composite electrodes aim to achieve softness and high conductivity to acquire a continuous high-quality biopotential. The composite electrodes are fabricated with various materials such as a polymer, fibers, and hydrogel, and that could be represented as soft materials for measuring biopotentials. We present the manufacturing method and characteristics of the composite electrodes with various materials. In the case of elastomeric composite electrodes, Lee et al. [5] reported soft, elastomeric composite elements for biopotential, as shown in Figure 3a. The elastomeric composites are made of three different types of carbon nanotubes (CNTs) (HANOS CM-95, CM- 250 and CM-280). In all cases, such as mechanical endurance, robustness, and deformation, CM-280 is an optimized composite material considering mechanical endurance, robustness, and deformation. Moreover, elastomeric composite electrodes based on CM-280 showed the lowest rate of electrical resistance changes among the three types of CNTs. From the signal acquisition quality perspective, such as the signal-to-noise ratio (SNR), the elastomeric composite electrodes based on CM-280 are comparable to commercial gel electrodes. This electrode is a representative electrode for overcoming the disadvantages of the existing electrode, such as skin irritation and dehydration. Lin et al. [38,84] designed conductive polymer foam electrodes based on urethane and taffeta materials coated with Ni/Cu on all surfaces (Figure 3b). This polymer electrode can reduce motion artifacts by absorbing the motion force and the rubbing and sliding of the electrode on the human skin. Fiber-type electrodes are generally divided into fabric-type and paper-type electrodes. As a flexible electronic, it has fiber-based substrate printability and is low cost, lightweight, and can be used in disabilities [88]. As shown in Figure 3c, fiber electronics manufacturing processes are also simple to apply in conductive inks on a fiber-based substrate. In previous research, Antti et al. [89] reported accessible silver-coated fiber-type electrodes (20 × 20 mm^2^). Fiber-based electrodes are affordable but are vulnerable to motion artifacts from the forehead depending on the facial movements.

To overcome the disadvantages of the previous fabric electrodes, Eskandarian et al. [90] introduced 3D-Knit fabric-type electrodes based on conductive elastomeric filaments (CEFs), which are flexible, breathable, and washable, as shown in Figure 3d. The conductive elastomeric materials are knitted or weaved to be electrodes, and the fabric electrodes also can be integrated into the general garment. This unique combination of fabric-type electrodes and garments enables one to monitor electrophysiological signals. The fabric-type electrodes are developed with a 3D structure to be compatible with human skin. The following is a brief summary of the manufacturing process. (1) CEF fiber is used for the electrode’s surface. (2) The polyester yarn is then knitted as a 3D structural filler in the spacer layer. To support the 3D structures, polyester is knitted on the back layer. With these fabric-type electrodes, smart garments can be used for the long-term monitoring of electrophysiological signals without severe levels of motion artifacts. Paper-based electrodes have similar advantages to the above fabric-type electrodes but have a simpler manufacturing process. Paper-based electrodes are fabricated using inkjet printing [91,92], spin coating [93], and screen-printing [94]. However, Golparvar et al. [57,95,96] introduced wearable graphene textiles with a different fabrication process [57]. First, an ordinary textile is dipped in a graphene oxide (GO) solution. Moreover, thermal treatment and chemical reduction are conducted to obtain reduced graphene oxide (rGO). These graphene textile electrodes promise flexibility, breathability, and usability for a daily garment. The flexibility is able to match skin deformations. Moreover, permeability relieves skin irritation relative to air and moisture. Due to usability, the wearable graphene textile electrode is likely to be adopted by sportswear companies for smart wearable devices.

Fiber-type electrodes have limited stretchability, which is not suitable for uneven skin. Moreover, those fiber-type electrodes are vulnerable relative to temperature and humidity. Some research groups presented hydrogel electrodes to overcome the limitation of the fiber-type electrode [61,97,98] (Figure 3e). Among those research groups, Wang et al. [98] introduced a conductive nanocomposite network hydrogel fabricated by projection microstereolithography (PμSL)-based 3D printing. This 3D-printed hydrogel shows high stretchability with high conductivity. Moreover, it can capture biopotentials precisely. As shown in Figure 3f, the 3D-printed hydrogel is stretchable and bendable even at low temperatures (−115 °C). Wan et al. presented the starch hydrogel patch made by lotus rhizome. As shown in Figure 3g, this conductive starch hydrogel has high stretchability (790%), adhesion, and a low Young’s modulus (4.4 kPa). This starch hydrogel patch enables a conformal attachment on uneven human skin based on these properties. To fabricate a starch hydrogel patch, skeleton material (lotus rhizome) and electrolyte (NaCl) are integrated. These materials allowed capturing EOG signals with biocompatibility and biodegradability. Wang et al. introduced another flexible hydrogel electrode [61], providing exceptional breathability, a low modulus (286 kPa), and adhesion to the human skin as a biocompatible biosensor. Compared to conventional gel electrodes, this hydrogel electrode has biocompatibility, which causes fewer skin irritations. This flexible hydrogel electrode is made of conductive hydroxypropyl cellulose/Polyvinyl alcohol (HPC/PVA) hydrogel and flexible polydimethylsiloxane (PDMS) substrate.

#### 2.1.2. Dry Electrodes

**Figure 4:**
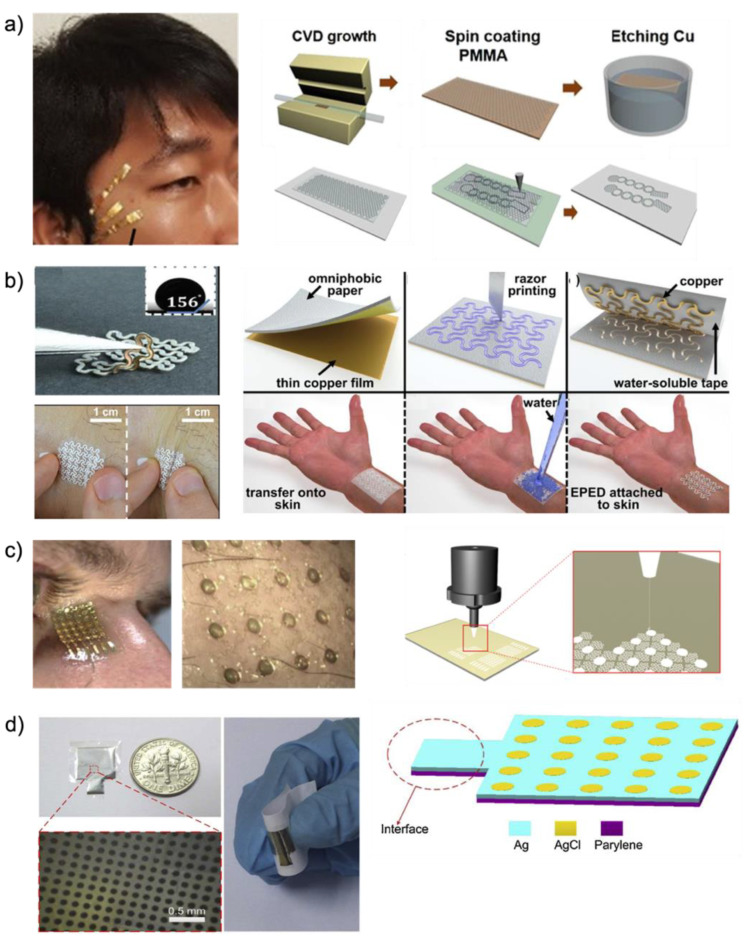
Schematic diagrams and images of micro-patterned electrodes: (**a**) Graphene electrode fabrication process based on a polymer material (reprinted under terms of the CC-BY license [11]. Copyright 2018, the authors. Published by Springer Nature). (**b**) Copper electrode fabrication process based on paper substrate (reprinted under terms of the CC-BY license [99]. Copyright 2018, the authors. Published by MDPI). (**c**) AgNPs electrode fabrication process via aerosol jet printing (reprinted under terms of the CC-BY license [30]. Copyright 2020, the authors. Published by Science). (**d**) Flexible dry Ag/AgCl electrode fabrication process via screen printing (reprinted with permission [100]. Copyright 2016 Elsevier).

Recent advancements in microfabrication technologies opened the possibility for micro-patterned electrode designs and facilitated the design of sophisticated micro- or nano-scaled electrode with diverse sizes, shapes, and even mechanical and electrical properties. Here, we introduce micro-patterned electrodes on various substrates such as polymer [11], paper [99], and metal (gold [1] and silver [100]). Among the three types of dry electrodes utilized for capturing EOG signals, the polymeric substrate was regarded as an attractive material because its scaleable property enables various forms of electrode fabrication. As shown in Figure 4a (left), Ameri et al. [11] introduced graphene electronic tattoos with ultrathin, ultrasoft, transparent, and breathable substrates. These electrodes are manufactured with graphene and polymethyl methacrylate (PMMA). Figure 4a (right) shows a manufacturing process; graphene is grown on copper foil, and the 350 nm film of PMMA is coated on graphene by spin coating. Then, the copper layer is etched away and rinsed with deionized (DI) water. The graphene/PMMA layer is transferred onto a commercial tattoo paper. Then, the graphene/PMMA layer on tattoo paper is carved with the shadow mask and a mechanical cutter plotter (Silhouette America Inc., Lindon, UT, USA). This electrode is designed in serpentine-shaped ribbons to enable stretchability (50%) [10]. Other electrodes are applied as paper-type substrates to materialize a dry electrode [99]. Epidermal paper-based electronic devices (EPEDs, Figure 4b (Left)) are manufactured by a benchtop razor printer, which is simple, low-cost, and compatible. As shown in Figure 4b (Right), to manufacture EPEDs, paper substrates are used. These paper substrates are silanized with fluoroalkyl trichlorosilane for inexpensive, water-resistant, and mechanically compliant materials relative to human skin. Moreover, conductive inks or thin films are attached to the side of paper substrates. The open mesh serpentine layout of the EPEDs is craved by a programmable razor printer (Silhouette CameoTM, Silhouette America Inc., Lindon, UT, USA). The silanization with fluoroalkyl trichlorosilane prevents the EPEDs from being wet because silane is used for hydrophobic paper. Due to the low thickness, the EPED is able to be compatible with skin wrinkles [101]. Moreover, the mechanical reinforcement of EPEDs allows withstanding accidental stresses of up to 2.5 MPa. The design of the EPED electrodes, the serpentine pattern, enables them to endure stretching up to 58% before mechanical failure. The “skin-like” bioelectrode made of metal (gold or silver) is feasible to draw the advantage of mesh-patterned dry electrodes (Figure 4c (Left)). One of the representative examples utilizing Au was introduced by Mishra et al. [1]. A cleaned glass slide is coated with primer (MicroChem Corp., Westborough, MA) for adhesion. After coating with PMMA and PI on the glass slide, curing of PMMA and PI is followed. Au deposition on the PI and photolithography-defined patterns is designed according to a “skin-like” fractal pattern. From the perspective of skin assessments, the fractal bioelectrode is advantageous over the conventional gel electrode. The conventional gel electrode causes skin irritation by heating skin temperature, while this “skin-like” fractal electrode shows a negligible change in skin temperature. This fractal electrode demonstrates mechanical compliance in both stretchability (30%) and bendability (up to 180°). Another manufacturing method of “skin-like” bioelectrode is aerosol jet printing (AJP) as shown in Figure 4c (Right). As a potentially low-cost and scalable printing method [102], AJP allows the direct printing of an open-mesh structure onto a soft membrane without using an expensive nano/microfabrication facility [103]. With silver nanoparticles (AgNPs) (UT Dots Inc., Champaign, IL, USA), AJP allows the direct printing of an open-mesh structure onto a soft membrane. The “skin-like” bioelectrode designed by computational modeling showed highly flexible (180° with a radius of 1.5 mm) and stretchable (up to 100% in biaxial strain) characteristics. Peng et al. [100] proposed a flexible dry electrode with an Ag pad and ten thousand micro-AgCl pads (Figure 4d). This flexible dry electrode is manufactured with parylene C (PC) (Sigma–Aldrich, St. Louis, MO, USA). As shown in Figure 4d (Right), the parylene layer is deposited on a glass wafer by chemical vapor deposition (CVD). After that, a positive photoresist (PR) spun on the parylene film is patterned by ultraviolet (UV) light. Next, a sputtering process and a lift-off process are carried out. Ag is electroplated and partly chloridized by electrochemical methods. Finally, the PR is removed. These dry electrodes are based on parylene, which is biocompatible, flexible, and good adhesive. Because this electrode is thin and flexible compared to conventional electrodes, it can maintain a stable and low electrode–skin impedance.

**Table 1:** Summary of electrodes for measuring EOG signals.

Electrode Type	Conductive Material	Supporting Substrate	Biopotential	Biocompatibility	Stretchability	Bendability	Fabrication	Size	Modulus	Advantage	Refs.
Polymer	CNT	PDMS	EOG, EEG	O	O	O	Mix and curing	20 × 5 × 5 mm^3^	Elasticity 4 MPa	Less changes in electrical resistance against mechanical deformation -High signal-to-noise ratio	[5]
	Ni/Cu	Urethane foam	EOG, EEG	O	X	X	Assembling Metal and foam	14 × 8 × 8 mm^3^	Compression set 5%	Low interference from skin-electrode interface	[38,84]
	Ag/AgCl	Parylene	EOG, EEG, EMG	O	X	O	Microfabrication process	10 × 10 × 0.05 mm^3^	-	Ease of thickness control, ultrathin fabrication Well-fitting skin topology	[100]
	Graphene	PDMS	EOG	O	50%	O	APCVD and Coating	6 × 20 mm^2^	-	Ultrathin, ultrasoft, transparent, and breathable. Angular resolution of 4° of eye movement	[11]
Fiber	Graphene	Cotton textile fabrics	EOG	O	X	O	Simple pad-dry technique	35 × 20 mm^2^	-	Simple and scalable production method	[104]
	Graphene	Textile fabrics	EOG	O	O	O	Dipping and thermal treatment	30 × 30 mm^2^	-	Possibility and adaptability for mass manufacturing	[42,57,96]
	Silver	Textile fabrics	EOG, EMG	O	X	O	Embroidering	20 × 20 mm^2^	-	Comfortableness and the usability with the measurement head cap	[89]
	CEF	CEF fibers	EOG, ECG	O	258.12%	O	Industrial knitting machine	20 × 20 mm^2^	Stress 11.99 MPa	Flexible, breathable, and washable dry textile electrodes Unrestricted daily activities	[90]
	Silver polymer	Escalade Fabric	EOG, EMG	O	O	O	Screen and Stencil printing	12 × 12 × 1 mm^3^	-	Textile compatible, relatively low cost for a production lineSmaller scale manufacturing	[105,106]
	Copper	Omniphobic paper	EOG, ECG, EMG	O	58%	O	Razor printer	20 × 15 mm^2^	Stress 2.5 MPa	Simple, inexpensive, scalable, and fabrication Breathable Ag/AgCl-based EPEDs	[99]
	silver/polyamide	Fabric	EOG	O	O	O	Mix and coating	10 × 10 mm^2^	-	Reduction in noise by appropriate contact	[40]
Hydrogel	PEGDA/AAm	-	EOG, EEG	O	2500%	O	PμSL-based 3D printer	15 × 15 mm^2^	-	Excellent stability and ultra-stretchability	[98]
	Starch	Sodium chloride	EOG	O	790%	O	Gelation process	30 × 10 mm^2^	4.4 kPa	Adhesion, low modulus, and stretchability No need for crosslinker or high pressure/temperature	[61]
	HPC/PVA	PDMS	EOG	O	20%	O	Coating	30 × 10 mm^2^	286 kPa	Well-adhered to the dimpled epidermis	[97]
	MXene	Polyimide	EOG, EEG, ECG	O	O	O	Mix and Sonicating	20 × 20 mm^2^	-	Low contact impedances and excellent flexibility	[107]
	PDMS-CB	-	EOG	O	O	O	Mix and deposition	15 × 15 mm^2^	2 MPa	Continuous, long-term, stable EOG signal recording	[108]
Metal	Silver	Polyimide	EOG	O	100%	O	Microfabrication process	10 × 10 mm^2^	-	Highly stretchable, skin-like, and biopotential electrodes	[30]
	Gold	Polyimide	EOG	O	30%	O	Microfabrication process	15 × 10 mm^2^	78 GPa	Comfortable, easy-to-use, and wireless control	[1]

### 2.2. Examples of Platforms for EOG Monitoring

From the wearable EOG device user’s requirement, which enables long-term comfort, research groups designed various types of wearable EOG device platforms. As shown in Figure 2e, previous EOG devices were bulky and many wires were attached, causing limitations with respect to the long-term or continuous monitoring of the user’s daily eye movements and inconveniences when incorporated the device into one’s attire. The contact between soft human skin and rigid EOG devices causes limitations such as noise during the collection of the biopotential [109,110,111]. With recent advances in wearable technologies, Yeo et al. [1,30] suggest that wearable sensor systems should be soft, compact, and built-in to solve the above problems [112]. In addition, researchers and subjects indicated that wearable sensor systems should not interrupt daily behavior [112]. Advances in circuit systems enable the wireless, real-time, continuous detection of biopotentials [113]. This section introduces four types of wearable platforms: glasses, face masks, headbands, and earplugs (Table 2).

#### 2.2.1. Eyeglass Type

**Figure 5:**
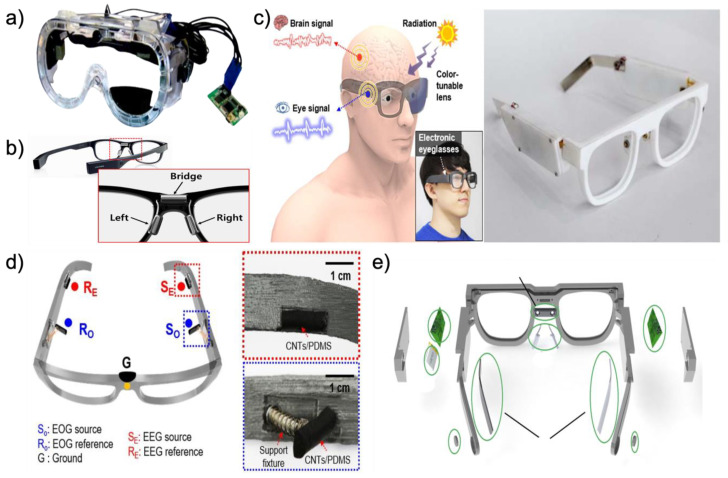
Examples of eyeglasses with electrodes. (**a**) Goggle type of EOG device (reprinted with permission under the terms of the CC-BY license [38]. Copyright 2021, the authors. Published by MDPI). (**b**) Eyeglass type of commercial EOG device (reprinted with permission [119]. Copyright 2016 ACM). (**c**) Eyeglass type of 3D-printed EOG devices (left: reprinted under terms of the CC-BY license [11]. Copyright 2018, the authors. Published by Springer Nature, right: reprinted with permission [32]. Copyright 2019 ACM). (**d**) Positions of CNTs/PDMS electrodes. (**e**) Positions of dry metal electrodes (left: reprinted under terms of the CC-BY license [11]. Copyright 2018, the authors. Published by Springer Nature, right: reprinted with permission [32]. Copyright 2019 ACM).

Glasses-type platforms enable convenient and inconspicuous applications and minimize user distractions with respect to autonomous long-term usage in daily life. As another advantage, the glasses-type platform can be used with prescription lenses because the glasses-type EOG devices are embedded within a traditional glasses frame [114]. Among those who wear glasses because of their eyesight, 92% of populations over 70 already wear glasses [115]. For the above reasons, these glasses-type platforms are likely to be adopted by elderly individuals who already require corrective eyeglasses [115]. Among the various glasses-type platforms, we introduce goggle-based devices, commercial devices, and devices manufactured by 3D printers. Figure 5a shows a goggle-based wearable EOG device aimed at applications such as mobile with activity recognition and context recognition. The goggle-based platform is designed to achieve the above aims with a user-friendly fit. Compared to the existing bulky devices as shown in Figure 2c,d, the weight of the entire device (i.e., including the goggles and circuit boards) is only 150 g and flat metal electrodes (Figure 2d) are placed around the user’s eyes through constant pressure. This comfort allows long-term wear to be used continuously for more than a few hours. Andreas et al. [116] manufactured goggle-type devices and predicted that mobile applications can be used to map a large TV as the input medium [38]. One of the commercial devices, the JINS MEME (JINS MEME Inc., Tokyo, Japan) eyewear, looks similar to a typical pair of glasses. To collect EOG biopotential with kinematic motion data, the JINS MEME has consisted of three metal electrodes, an accelerometer, and a gyroscope. Three metal electrodes are placed on the bridges and nose pads of the glasses to acquire EOG signals in the horizontal and vertical dimensions. The accelerometer and gyroscope are embedded in one of the arms of the glasses to collect motion data. These embedded sensors and metal electrodes can real-time, continuously detect human activity data. JINS MEME eyewear is shown in Figure 5b [39]. With the recent development of 3D printer technology, Lee et al. [5] and Kosmyna et al. [117] are directly manufacturing wearable platforms in the form of glasses, as shown in Figure 5c. Here, we introduce multifunctional electronic eyeglasses (E-glasses) made using a 3D printer. In wireless, real-time modes, these 3D-printed eyeglasses can monitor biopotentials such as EEG, EOG, and UV intensity. Instead of conventional gel electrodes, soft conductive composite electrodes are placed on E-glasses for electrical and mechanical superior properties. The device is designed to maintain seamless contact between skin and electrodes through constant pressure for reliable biopotential measurements. Various human motions also can be observed by analyzing the accelerometer. As one of the advantages of the glasses-type platform, this device can transform the lens required by the user, such as sunglasses for the UV protection [31,118,119], or prescription lenses for eyesight. As shown in Figure 5d, details on electrodes for recording biopotential signals such as EOG and EEG are listed (SO: source electrode for EOG; RO: reference electrode for EOG; SE: source electrode for EEG; RE: reference electrode for EEG; G: ground electrode). It is possible to apply constant pressure to the CNTs/PDMS electrodes through the E-glasses legs and supports fixture [5]. Figure 5e shows that another 3D-printed glasses-type platform consists of two printed circuit boards (PCBs), two EEG electrodes, two EOG electrodes, a reference electrode, and a lithium polymer (LiPo) battery. This device is made of nylon plastic, which is a flexible material. The particular parts of the eyeglasses frame are made of silver as electrodes to monitor EOG and EEG. The EOG electrodes are located on the nose pad similar to the E-glasses structure above. Moreover, an extra silver electrode is placed on the nose bridge of the glasses to serve as a reference electrode (EOG electrodes (1), reference electrode (2), EEG electrodes (3), PCBs (4), LiPo battery (5), and small open chamber for piezoelectric element to deliver bone-conducted sound) [32].

**Table 2:** Summary of wearable EOG platforms.

Wearable Platforms	Electrodes			Platforms		Refs.
	Types	Materials	Counts	Size	Features	
Earplug	Foam	Silver	2	2 × 2 × 1 mm^3^	-Stable and comfy during sleep	[44,45]
	Foam	Conductive cloth	2	2 × 2 × 1 mm^2^	-Stable and comfy during sleep	[43]
Eyeglass	Gel	Ag/AgCl	6	15 × 14 × 5 cm^3^	-Lots of wires were attached	[37]
	Metal	Silver	3	15 × 14 × 5 cm^3^	-Real-time delivery of feedback in the form of an auditory	[32,33,117]
	Metal	Ag/AgCl	5	15 × 14 × 7 cm^3^, 150 g	-Constant pressure for electrodes	[34,116]
	Foam	CNT/PDMS	5	15 × 14 × 5 cm^3^	-UV protection via sunglass lens	[5]
	Foam	Ni/Cu	5	14 × 12 × 7 cm^3^	-Absorbing the motion force via Foam and platform	[38]
Facemask	Fiber	Silver/Polyamide	3	14 × 7 × 2 cm^3^	-The wires are embedded in the eye mask platform	[40]
	Metal	Silver/Carbon	8	20 × 15 cm^2^	-Tattoo-based platform-Stable and comfy	[41]
	Fiber	Graphene	5	15 × 7 × 2 cm^3^	-High degree of flexibility and elasticity	[42]
Headband	Gel	Ag/AgCl	4	15 × 7 cm^2^	-Waveforms were well measured on the headband platform	[51]
	Metal	Ag/AgCl	4	15 × 7 cm^2^	-Reduction in the total cost by using disposable Ag/AgCl medical electrodes	[55]
	Fiber	Graphene	3	15 × 7 cm^2^	-Long-term EOG monitoring applications	[21,57,96]
	Fiber	Silver	5	15 × 7 cm^2^	-Reusable and easy-to-use electrodes are integrated into the cap.	[89]
	Fiber	silver-plated and nylon	3	15 × 7 cm^2^	-Long-term EOG monitoring applications	[58]

#### 2.2.2. Facemask Type

**Figure 6:**
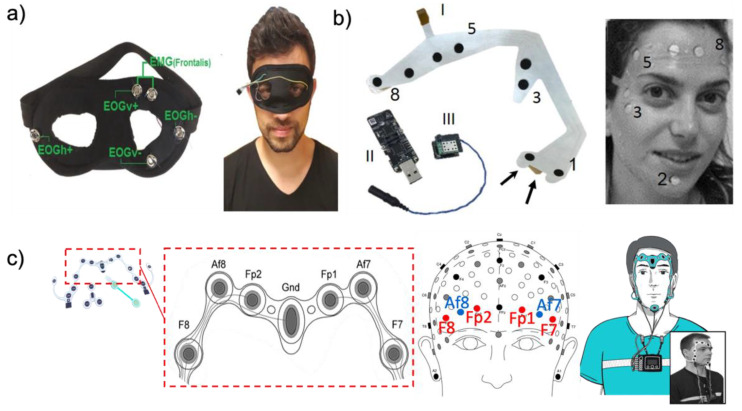
Examples of facemask platforms for EOG monitoring. (**a**) Face type of EOG device with graphene-coated tissue electrodes (reprinted under terms of the CC-BY license [42]. Copyright 2019, the Authors. Published by JAIC). (**b**) The electrode array system and a subject wearing a temporary-tattoo eight-electrode array (reprinted with permission [41]. Copyright 2019 IOP). (**c**) Positions of a screen-printed electrode set and a subject wearing screen-printed electrodes (reprinted with permission [7]. Copyright 2019 Elsevier).

For a comfortable and stable fit, a face mask-type platform was presented. Among the various face mask type platforms, we introduce different types of eye masks (Figure 6a) as well tattoo-based and commercial devices. In the case of the eye mask platform [40], electrodes are made of conductive sponge materials. Three dry sponge electrodes are placed on the eye mask around the user’s eyes. Two electrodes are used to acquire the EOG signal, and the other one is used as a reference electrode. To reduce the pressure applied to the skin, the manufactured eye mask platform fits the shape of the skin deformation. The wires are embedded in the eye mask platform to reduce noise by the movement of the wire. Another eye mask-type platform integrates a sleep eye mask with electrodes. This eye mask platform uses EXCELLENT 47 (Moxie Corporation, Taipei, Taiwan) instead of a conventional gel electrode. The proposed dry fabric electrode consists of a high-performance silver/polyamide (20%/80%) compound. The combination of the sleep eye mask and the soft fabric electrode enables a reduction in noise by appropriately contacting the user’s skin to acquire a biopotential. Another face mask platform reported by Shustak et al. [41] is a tattoo-based EOG device as shown in Figure 6b. This tattoo-based device acquires various biopotentials such as EEG, EOG, and EMG using a dedicated electrode layout on the user’s face. This electrode layout is implemented on thin polyurethane films with silver electrodes coated by a bio-compatible C layer. To contact between skin and a tattoo-based platform, a double-sided adhesive is used for a stable attachment [120]. The position of the electrodes is shown below with a number and acquired biopotential: EMG electrodes (1 and 2), EOG electrodes (3 and 4), and forehead EEG electrodes (5~8). The Nox A1 portable H-PSG system (Nox Medical, Reykjavík, Iceland) together with an ambulatory electrode set is a face type of commercial device that can capture EOG and EEG signals. As shown in Figure 6c, EOG electrodes (F8 and F7) and EEG electrodes (Af8, Fp2, Fp1, and Af7) are placed on the forehead [121].

#### 2.2.3. Headband Type

**Figure 7:**
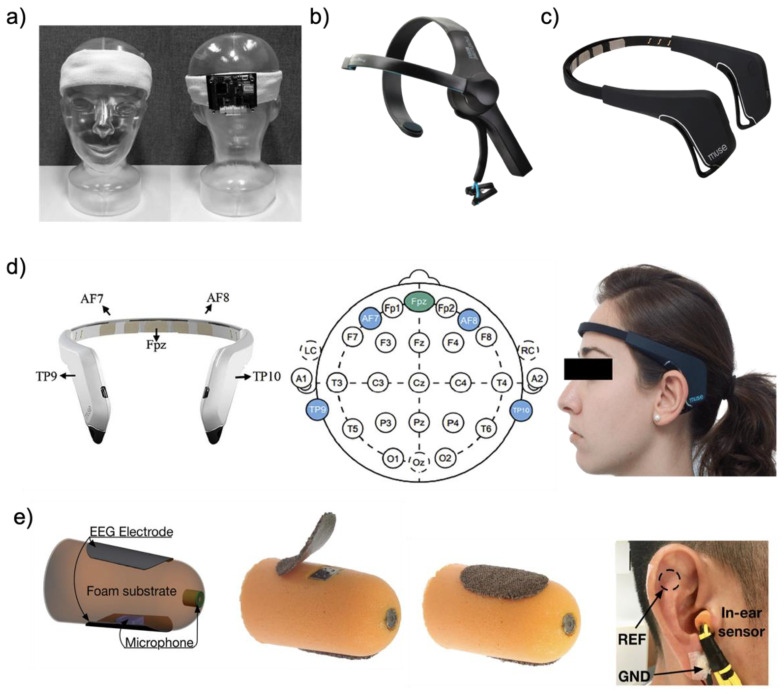
Examples of headband platforms and earplugs with electrodes. (**a**) Example of headband type of EOG device. (Reproduced under terms of the CC-BY license [51]. Copyright 2017, the Authors. Published by MDPI). (**b**,**c**) Headband type of commercial EOG devices. ((**b**): Reprinted with permission [4]. Copyright 2019 Elsevier, (**c**): Reprinted under terms of the CC-BY license [123]. Copyright 2017, the Authors. Published by MDPI). (**d**) Position of embedded dry electrodes with the subject wearing a commercial device. (Left: Reprinted with permission [124]. Copyright 2019 Elsevier, Middle and Right: Reprinted under terms of the CC-BY license [123]. Copyright 2017, the Authors. Published by MDPI). (**e**) Earplugs type of EOG device. ((**e**): Reprinted under terms of the CC-BY license [6]. Copyright 2017, the Authors. Published by Springer Nature).

EOG signals can be sufficiently acquired not only around the eyes but also on the forehead. Heo et al. [51] designed a wearable EOG device based on a headband to acquire the forehead EOG signal. We introduce soft fabric headband-type and commercial devices among the various headband-type platforms. In general, dry electrodes are placed around the forehead inside the headband. The two electrode sets are prepared to measure horizontal and vertical eye movements, and the other one is used as a reference electrode. As shown in Figure 7a, the printed circuit board (44 mm × 55 mm) is placed on the back side of a headband. Such a soft fabric headband-type platform can provide a comfortable fit and can stably secure the electrodes on the human skin. One of the commercial headband types of wearable devices (Figure 7b), NeuroSky (San Jose, CA, USA) is used for brain–computer Interface (BCI) equipment [49]. The NeuroSky headband is adjustable and requires low costs, with an inexpensive dry sensor. Since one dry electrode located on the forehead acquires a biopotential, there is not enough information contained in the EOG signal with EEG signal, but it includes built-in electrical noise reduction software/hardware, making it easy to detect the EOG signals with the EEG signal. Another commercial headband-type wearable device, Muse, has four biopotential channels for monitoring eye movements and brain waves. Moreover, this device has a three-axis accelerometer and gyroscope for detecting head motion. In the case of the Muse device (Figure 7c), the electrodes are located on the forehead and behind the ear (as shown in Figure 7d two on the forehead (AF7 and AF8) and two behind the ear (TP9 and TP10)), with the reference electrode located at the center of the forehead (Fpz) [122,123].

#### 2.2.4. Earplug Type

This earplug-type platform aims to be a human-centered, compact, non-obtrusive, and ergonomic wearable device. In addition, because it is non-invasive, users can use it for a long time without fatigue. Figure 7e shows that a pair of small and thin passive electrodes are attached to the surface of the earplugs [6]. Alternatively, another style of earplug-type platform uses an electrode that is made from a small piece of conductive silver cloth layered by pure and thin silver leaves many times on top. This wearable platform enables the earplugs to overcome the delicate structure of the human ear and users can use it comfortably inside the ear when sleeping. To ensure a comfortable and snug fit, the substrate material of the earplug-type platform is a memory foam that absorbs artifacts stemming from small and large mechanical deformations to the ear canal’s walls. The placement of an earplug-type platform should properly be placed to acquire the EOG signal with the EEG signal. The suggested place is the main electrode in one ear and the reference electrode in another.

### 2.3. Signal Processing Algorithms and Applications

#### 2.3.1. EOG Signal Processing

**Figure 8:**
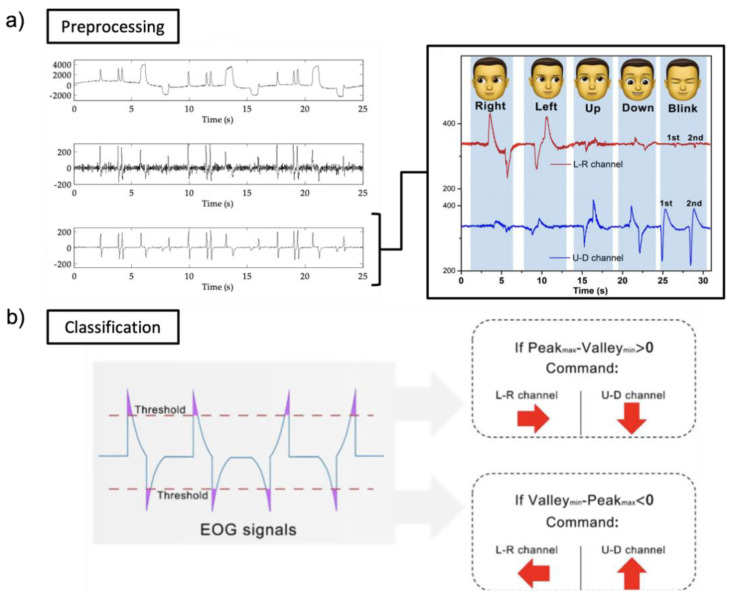
Signal processing and data analysis. (**a**) Recorded EOG signals depending on eye directions (Left: Reproduced under terms of the CC-BY license [51]. Copyright 2017, the Authors. Published by MDPI, Right: Reproduced with permission [61]. Copyright 2021, Wiley-VCH GmbH). (**b**) Schematic algorithm diagram using threshold (Reproduced with permission [61]. Copyright 2021, Wiley-VCH GmbH).

Figure 8a shows the detailed pre-processing with EOG signals received through Bluetooth low energy (BLE) embedded in the circuit (Sample rate of 250 Hz). Before classification, noise and baseline drift removal and data averaging are implemented as pre-processing. A band-pass filter is applied to remove noise components [125]. When the received EOG signal (analog) from the skin is converted into a digital value, a DC offset is generated. The first DC offset value is removed from all signal values to remove drift (DC offset). Noise and trends can sometimes interfere with data analyses and should be eliminated. To smooth the EOG waveforms, samples are divided into minimal sets and averaged. It is used as a method for removing noise. EOG signals are generally classified in five directions (left, right, up, down, and blink). To classify into five classes, thresholds are setup with a specific value (horizontal channel: right (400 μV) and left (−400 μV); vertical channel: up (400 μV), down (−400 μV), and blink (500 μV)). In other ways, EOG signals are classified by comparing the amplitude or wavelength of the peak, or whether the difference from the peak to the peak is negative or positive, as shown in Figure 8b. However, signal processing alone cannot detect the class much. Moreover, medical analyses have many limitations when using signal processing. Here, we introduce machine learning for more classes or medical analyses.

#### 2.3.2. Machine Learning

**Figure 9:**
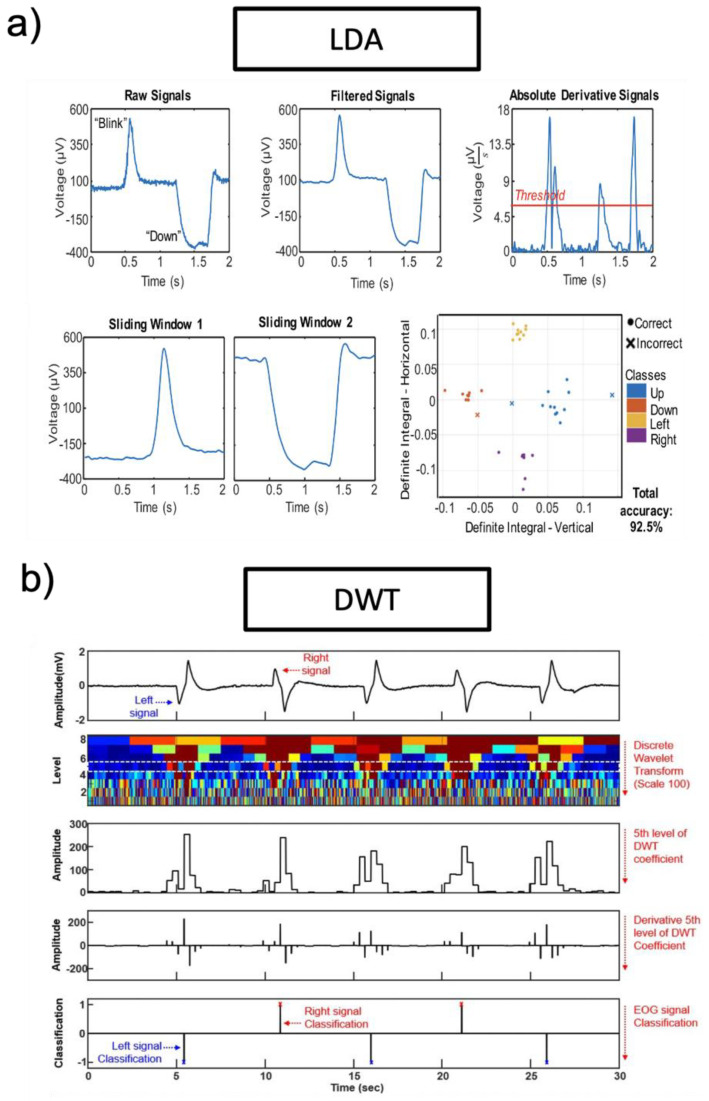
Machine learning for data analysis (**a**) Signal processing sequence with an LDA classifier (Reprinted with permission [1]. Copyright 2017, Elsevier B.V.). (**b**) Signal processing sequence with a DWT classifier (Reprinted with permission [5]. Copyright 2020, American Chemical Society).

Recently, research groups introduced machine learning to analyze EOG signals. Machine learning technologies are applied according to the purpose of each study and application. However, different machine learning technologies can be used for the same purpose. Researchers introduce a discrete wavelet transform (DWT) classifier and a linear discriminant analysis (LDA) classifier among machine learning technologies. LDA is a common classifier, which uses dimensionality reduction techniques in machine learning. This classifier can solve two-class classification problems. Figure 9a is an example of an LDA classifier (targeted EOG from eye movements of “blink” and “down”). To remove noise, a third-order bandpass filter (Butterworth) is used. By using thresholds, a series of peaks were detected. The start time and end time detected by the threshold are factors that increase detection accuracy. Pre-processed EOG signals are divided into test data sets and training data sets. Test data sets and training data sets are transferred to the LDA classifier, as shown in Figure 9a. The LDA classification plot includes both correct (o) and incorrect (x) classes. Another machine learning technology is DWT which is one of the wavelets transforms. The wavelets are sampled at discrete intervals. As shown in Figure 9b, the DWT classifier targeted EOG from eye movements of “left” and “right”. The acquired EOG signals are classified based on eye movements with an angle of eye rotation. The fifth level of DWT coefficient with a scale of 100 and the “sym8” basis function is selected for DWT performances. To remove noise, a third-order bandpass filter (Butterworth) is used (fc = 0.5−50 Hz).

#### 2.3.3. Applications

**Figure 10:**
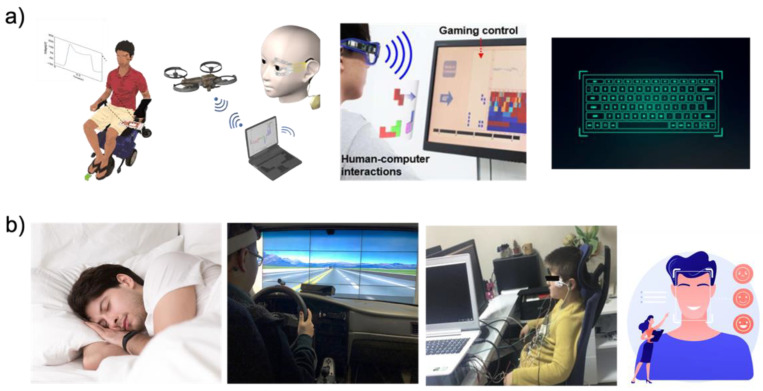
(**a**) Controller-type applications such as wheelchairs, drones, game interfaces, and virtual keyboards (1st: reprinted with permission [1]. Copyright 2017, Elsevier B.V., 2nd: reprinted under terms of the CC-BY license [11]. Copyright 2018, the Authors. Published by Springer Nature, 3rd: reprinted with permission [5]. Copyright 2020, American Chemical Society, 4th). (**b**) Healthcare monitoring systems applications and medical health status analyses applications (2nd: reprinted with permission [63]. Copyright 2020, Elsevier B.V., 3rd: reprinted with permission [65]. Copyright 2020, Walter de Gruyter GmbH).

With the recent development of wearable EOG device platforms, EOG signals can be easily acquired and applied to HMI applications without limitations from previous bulky and wired EOG devices. The use of HMI applications is increasing rapidly. There are two types of applications, such as the controller type and analysis type as shown in Table 3. As shown in Figure 10a, in the case of the controller type such as wheelchairs [1,4,51,52], drones [11,59], game interfaces [5,36,47,60,61], and virtual keyboards [34,38,51,62], a command is put into the HMI by detecting the direction of the eye. However, the EOG signal for HMI has eye angle and gaze detection limitations. In general, four or six eye directions can be detected by signal processing. The limited number of eye movement detection is limited for HMI applications, which require complex commands. The EOG signal is sensitive to noise and users’ small movements. Therefore, there is a limit to being applied to surgical robots that require accurate movement. To overcome the above limitations, research groups are simultaneously analyzing biopotentials. Figure 10b shows various healthcare monitoring systems [7,40,41,44,45,63], and medical health status analyses [64,65,66] have been conducted using both biopotentials, such as EOG, EEG, and EMG, with signal processing. In general, EMG, EOG, and EEG signals are simultaneously obtained from the subject’s face, and information for healthcare analyses is obtained via signal processing with machine learning. The field that received a lot of attention is sleep or fatigue monitoring analyses. To monitor the sleep stage, Shustak et al. [41] recorded EMG, EOG, and EEG using a wireless system. This sleep monitoring system showed clear differentiation of the sleep stage for 6 h. This research group showed the potential of sleep disorders monitoring systems in the home environment by demonstrating sleep stage monitoring. Jiao et al. [63] presented a novel model for driver sleepiness detection by simultaneously analyzing EEG and EOG signals. The driver sleepiness detection system based on EEG and EOG is analyzed by the long-short term memory (LSTM) classifier, achieving a mean accuracy of 98%. The research group determined that a wearable sleepiness detection system could be used to reduce traffic accidents by detecting sleepiness. From a healthcare perspective, researchers are using EOG signals to analyze attention deficit hyperactivity disorder (ADHD) [64,65,66] or emotion detection [126,127,128]. Soundariya et al. [127] introduced emotion-recognizing systems based on EOG signals from eye movements. The recorded EOG signal is classified as happiness, sadness, anger, fear, and pleasure by the supporting vector machine (SVM) classifier.

## 3. Eye Trackers

Recent advances in computing power became powerful enough for real-time eye tracking, which allowed using video and screen-based eye trackers [67,129]. Since then, with new technologies in tracking optic cameras and machine learning processes, eye tracking has been widely utilized with stationary cameras or cameras embedded glasses [67,68]. These cameras can record corneal infrared light reflection for tracking pupil position, mapping the tracked gaze while recording, and calculating other parameters such as tracking rate, dwell time, and pupil dilation [68]. These parameters are used for dynamic stimulus analyses to create an eye concentration marker, which is essential in tracking various human stimuli and human applications [68,130]. Recent eye tracking technology proposed integration to virtual reality (VR) and mixed reality (MR) setups to fulfill the demand for the entertainment domain and cognitive functioning domain for clinical assessments [131,132,133,134,135].

### 3.1. Details of Eye Trackers

#### 3.1.1. Human Eye Movement and Stimuli

**Figure 11:**
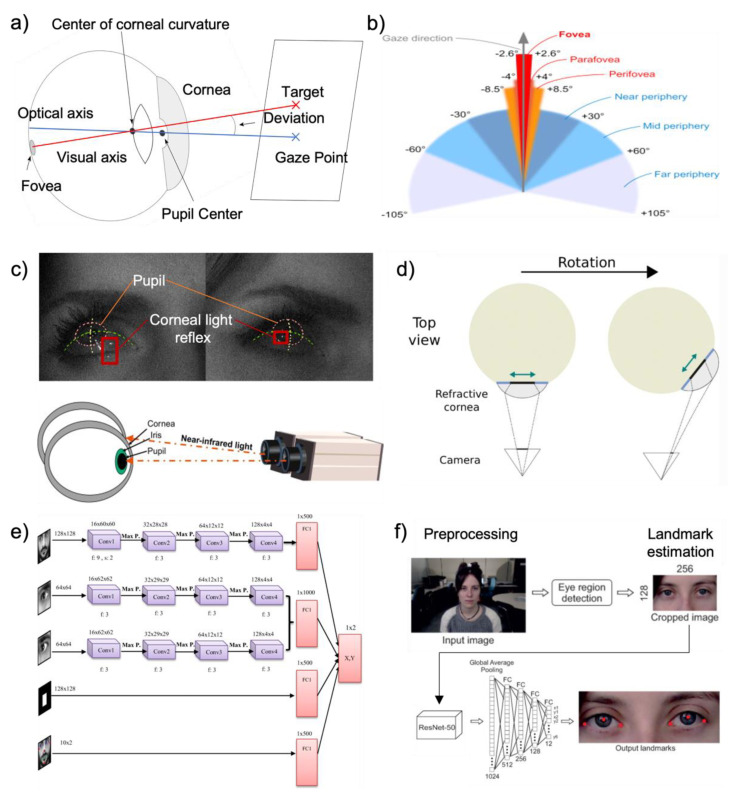
Eye movements and eye tracking technology. (**a**) Optical metric for human eye tracking (Reprinted with permission [146]. Copyright 2020, The Psychonomic Society, Inc.). (**b**) Eye foveal angle and human vision span (Reprinted under terms of the CC-BY license [131]. Copyright 2021, the Authors. Published by Elsevier Ltd.). (**c**) Eye grid and corneal light reflection in eye tracking systems (Reprinted under terms of the CC-BY license [135]. Copyright 2012, the Author. And Reprinted with permission [141]. Copyright 2014, Elsevier). (**d**) Illustration of relative cornea location between camera and eye, during eye rotation (Reprinted under terms of the CC-BY license [147]. Copyright 2021, the Authors. Published by MDPI). (**e**) Eye motion and gaze prediction model with CNN (Reprinted with permission [145]. Copyright 2022, Springer Nature). (**f**) Eye landmark estimation with image processing used for custom eye tracking solutions (Reprinted under terms of the CC-BY license [148]. Copyright 2021, the Authors. Published by MDPI).

All natural main eye movements are used to reposition the eye’s visual axis on the fovea [136]. The anatomy of the human eye is presented in Figure 11a. When the eye looks at a target, visual axis connects fixation point to center of the entrance pupil, front, and rear nodal point [137]. The eye moves when a user looks at an object to perceive stationary objects [136]. In real eyes, the fovea is displayed slightly inferior and temporally displaced from the point where the optical axis meets the retina and detects eye movement [137]. In general, the eye has six degrees of flexibility, three rotations, and three translations inside the eye socket [138,139]. The eye is rotated by two pairs of direct muscles that allow six degrees of freedom in eye movement control [67,136]. Eye movements can be classified into two main categories. First is saccadic movements. When we attempt to fixate the eye gaze to target area of interest, the eye does not stay still but continuously moves [131]. As known as rapid eye movements, saccade quickly adjusts visual axis of the eye on the fovea to interest area which is highly reflexive and voluntary [135]. The movement changes the eye’s vision to the object by gaze angle control [131]. Moreover, microsaccades (fixational saccades) are small eye movements that constitute fixation, which is the basis of visual perception [135]. The second category is for stabilizing movements, which attempt to hold the eye, or movement for a stable retinal picture [3,131,135]. Fixations occur when the gaze is fixed for a long time on a particular constrained area, providing fixational dynamics and statistics [136,138]. Figure 11b shows the foveal angle, and human vision span around the gaze direction; these numbers vary in different studies. While looking at an object with each eye’s fixation point remaining on the fovea, drift is an uneven and relatively slow movement of the eye’s axis [135]. The iris controls the amount of light admitted into the retina by contracting and expanding the pupil [136]. The crystalline lens, located behind the pupil, receives and focuses the image on the retina [136]. A transparent biconvex structure of crystalline lens controls focusing and accepting the image on the retina located behind the pupil [67]. The retina is next in control of converting the received image or visual stimuli into electric signals, and it transmit the visual cortex through optic nerves and stimulates the occipital lobes of the brain [133,138].

#### 3.1.2. Principles of Eye Tracking Technology

When detecting an eye, it is essential to differentiate the eye’s appearance because it can change depending on the angle that the user is observing [135]. Non-invasive Eye trackers rely on measurements of the eyes’ observable characteristics, including the pupil, iris-sclera boundary, and corneal reflection of nearby light sources [135,139]. As shown in Figure 11a,c, a technique based on corneal reflections measures the position of the corneal reflection of an infrared (IR) light reflected to the pupil that can track the gaze direction accurately [129]. The most widespread method for tracking eye movements is screen-based or uses video oculography, which includes reflection of iris and corneal or the pupil and corneal [136,140,141]. As Figure 11c illustrates, screen-based gaze tracking technologies are simple to use and set up for various applications [71,131,132,133,135]. The pupil and limbus information are the most often used features for tracking [138]. Tracking limbus, which is the boundary between sclera and iris, can trace horizontal eye rotations because of their contrast [67]. Monotonous limbus tracking systems have poor vertical precision because the eyelids partially obscure the iris [67,136]. The pupil is more challenging to track due to less contrasts between the pupil and the iris, but it can be distinguished when illuminated by an infrared light source on the camera axis with an on-axis light source [3,136,139]. This produces a “red-eye” effect image [3,136]. IR light sources are frequently used in eye trackers to increase the contrast between ocular features [138]. This is due to the fact that the IR is invisible and does not distract or interfere with the user when tracking [136,140]. With this unique characteristic, the eye tracker has been successfully integrated to head-mounted, wearable, and infrared-based gaze trackers [132,133,134,139]. The system consists of an optical camera, IR light sources, a CPU for data processing, and a screen or monitor to determine the subject’s eyes’ focus [135,139]. For accurate gaze location in a video-based system, high-resolution eye pictures are required [3,71,142]. Image processing is required to calculate the three-dimensional rotation angles of the eye, and these algorithms are used to determine the pupil location, cornea glint positions, and other properties of the eye [67,138,140], as shown in Figure 11d. The point and direction of gaze can be computed instantly by an eye tracker using low-cost cameras and image processing technology [131,138]. Recent developments in various machine learning techniques and algorithms have been made with an accuracy of under one degree [67,68,143]. Recent studies attempted to improve gaze data to predict accurate eye motions by presenting an end-to-end user-specific prediction model with convolution neural network (CNN) architectures [3,131], as shown in Figure 11e. With human–machine interfaces, the practical AI application begins with data collection, data cleaning, standardization, and then data interpretation using algorithms [144]. Deep-learning prediction models have overcome limiting factors in real-world conditions [145]. Hence, bioelectrical signals provide a natural and interactive way for humans and machines to connect and are extensively used in clinical diagnosis and rehabilitation with machine learning [144].

#### 3.1.3. Employment of Eye Tracking Technologies for Applications

Eye tracking is used to implement where and when the user’s eyes are focused [3]. The eye movements, such as saccades, smooth pursuit, vergence, and vestibulo-ocular movements, indicate human perception and recognition [136,140]. An improved sensor technology expands the possibility of a comprehensive understanding of a user’s visual attention [149]. Recent studies show that viewing emotionally toned or visual stimuli information is observed with an increased pupil size of the eyes, along with other features such as fixation duration, and saccades [149,150]. within addition to the pupil’s diameter, other variables such as fixation length, saccades, and EOG signals can also be used to identify emotions [3,139]. While eye tracking signals and information indicates the user’s behaviors, the system is widely used in human–computer interaction (HCI) and usability application research studies [3,69,141,142]. Moreover, a customized and personalized eye tracking system increases accuracy and allows more application in the areas of cognitive science, clinical assessment, and contents creation with affective information [70,142,149,151]. The development of eye trackers allows accurate eye tracking data to be integrated into a conventional clinical measurement system for higher brain functions such as cognition, social behavior, and higher-level decision-making measured by eye movement [70,152]. Eye movement data have been used by several research groups to distinguish patients with mental disorders such as schizophrenia or to examine eye movement traits that have a genetic component in relation to finding the risk of autism before the emergence of verbal-behavioral abnormalities [70,152]. Another study proposed a framework for vehicle control, which anticipates a driver’s real-time intention over future maneuvers by analyzing the gaze and fixation patterns of the driver [134]. Image processing and eye landmark estimation are the primary eye tracking technology used for control, as shown in Figure 11f. The study proposed future work for designing a customizable intention prediction model on vehicle control using strategy synthesis [134,153]. Recent eye tracking advances will significantly impact next-generation application solutions [69]. We will discuss these issues and related work in Section 3.4.

### 3.2. Eye Gaze and Movement Estimation

Many pupil center identification techniques have been presented in recent years using conventional gaze tracking with optical metrics, image processing, and machine learning-based techniques [69,70]. Previously, conventional methods are separated into two categories: optical modeling and characteristics modeling [136]. Optical modeling is used to calculate optical information mathematically and to examine the location between the angles of the input vectors and the location of intersection, which is computed as the pupil’s center [3,71,136]. Characteristic modeling estimates the pupil’s center by segmenting the pupil’s edge depending on its features in terms of contrast, contour, or color [136,142]. 

#### 3.2.1. Eye Tracking Techniques and Algorithm

Recent appearance-based algorithms [69,71,142] estimate the pupil center and feature with appearances when the subject focuses at a specific point in the scene. Since the method utilizes a computational approach, a large set of computing resources, including image dataset, processing power, and prior machine learning training, is required [154].

*PCCR—Pupil Center-Corneal Reflection and Bright and dark Pupil Effect*

**Figure 12:**
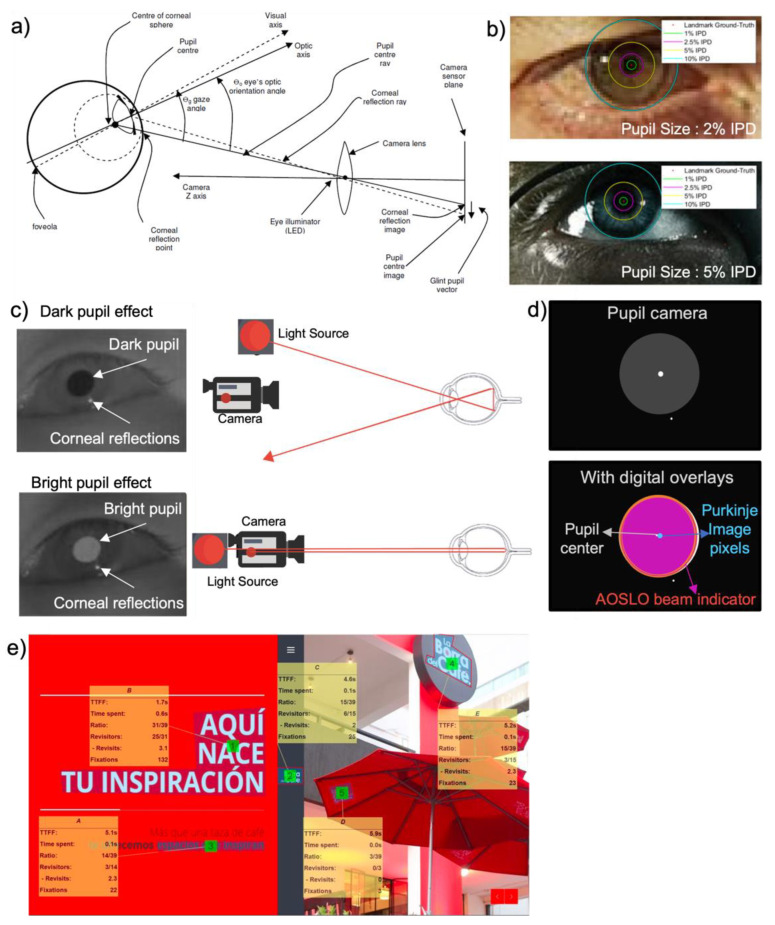
Eye gaze and movement estimation. (**a**) Optical metric for pupil center corneal reflection (PCCR) eye-gaze-tracking technology (Reproduced under terms of the CC-BY license [72]. Copyright 2013, the Authors. Published by ProQuest LLC). (**b**) Pupil images indicating inter-pupillary distance with two different landmark proportion (Reprinted under terms of the CC-BY license [148]. Copyright 2021, the Authors. Published by MDPI). (**c**) Dark and bright pupil effect and IR light source correlation with eye (Reproduced under terms of the CC-BY license [145]. Copyright 2022, the Authors. Published by Springer Nature). (**d**) Conversion of grey imagery and digital overlay indicator for pupil. (**e**) Multiple object eye movement analysis with time to first fixation (TTFF) (Reprinted under terms of the CC-BY license [157]. Copyright 2022, the Authors. Published by MDPI).

The PCCR method is one of the eye gaze tracking techniques to measure the direction of the eye’s gaze [155]. As shown in Figure 12a, the vector distance between the corneal reflection and the pupil center within the camera image can be used to calculate the eye’s orientation angle [72]. The line that connects the center of the camera lens and the center of the corneal sphere is utilized to measure both the vertical and horizontal elements of the eye’s orientation angle [72]. In the PCCR method, a single corneal reflection is utilized [71,72]. In the PCCR method, the corneal surface approximates a perfectly spherical mirror; thus, the vector from the pupil’s center to the corneal reflection within the camera image is closely related to the direction in which the eye is looking [72,73,135]. If the head is kept stationary while the corneal surface rotates, the glint remains stationary. By contrasting the corneal reflection and the pupil center, the eye tracking system can identify the direction of the gaze [71]. The corneal reflection is visible when a user stares directly at the camera close to the center of the pupil image [69]. When the user switches their attention upward from the corneal reflection, the pupil center shifts upward. Similarly, when attention is shifted downward, the glint–pupil vector points, and the pupil center moves downward [69]. Figure 12b shows the proportion of images with an error less than each percentage value of the inter-pupillary distance (IPD) with recent work on pupil center detection with CNN [148]. The result shows the proportion of images with an error less than each percentage value of the inter-pupillary distance and proposes possible limitations in tracking accuracies [148]. Inter-pupillary distances are expressed as a percentage of distances from the accurate eye pupil landmarks as shown in Figure 12b. Figure 12c demonstrates how the IR light source illuminates the user’s eye and creates two different pupil images and effects: bright and dark pupil [136]. For pupil detection and tracking, both bright and dark pupil effects are used [135,136]. A brighter pupil image can be created when using light sources parallel to the axis of the camera [135]. Since most of the light enters the eye along the optical axis and most of the light reflects back from the retina, this will cause the pupil to be brighter, which is called the “bright pupil effect” [72]. If the pupil is illuminated by light sources that are not parallel to the optical axis of the eye, it appears to be darker than its surroundings [141]. Since multiple corneal reflections and a variety of off-axis light sources produce darker pupil images, it is called the “dark pupil effect” [136]. The location of the illumination source and the camera’s optical axis determines how these two types of images differ from each other. When the light source is aligned coaxially with the optical path of the camera, the bright pupil image is created [136]. The eye then reacts as a retroreflector as the light reflects off the retina and creates the bright pupil effect. The pupil appears dark if the light source is located outside the camera’s optical axis because the retina’s retro-reflection is located away and creates the dark pupil effect [135,150]. Pupil contour extraction is a primary aspect of both feature extraction methods [135]. Due to the low contrast at the boundary between the pupil and iris, the pupil is difficult to distinguish in the eye [72]. Figure 12d shows researcher’s attempt to apply pupil tracking using grey level imagery and digital overlay indicators either of dark or bright pupils instead of employing threshold difference photos [73,148]. By overlapping the pupil between the images, the pupil image can detect directional movements accurately [136].

*Time to first Fixation and Object of interest*

The time to first fixation (TTFF) measures the speed at which respondents fixed their attention on an area of interest. TTFF is a simple but essential eye tracking metric [156]. Fixations are eye movements that naturally correspond to the intention and desire to keep one’s attention focused on a use interest point [72]. Fixation stabilizes the retina over a still object of interest, and the gaze remains on a certain region for an extended period of time [135,156]. TTFF measures how long a respondent can fixate on a particular area of interest (AOI) after the stimulus has started. TTFF can indicate the horizontal movement of stimulus-driven search. Fixations, which are still periods that happen in-between saccades on static scenes, are the major periods during which visual experience and recognition occur. Fixations are distinguished by small, high-frequency drifts and microsaccades that oscillate. Since the responders initially prefer to focus the center of the image over its edges, more bias toward the center occurs. This prevents the scene from being blind by preventing the image from fading [72]. The size and color of the objects in the AOI impact measuring TTFF. More distinguishing characteristics are frequently the subject of faster emphasis [72].

TTFF measures how quickly a target is identified and quantifies the attention; the shorter the TTFF, the greater the target’s visual significance [73]. The fixation duration is between 200 and 600 milliseconds, and the image formed on the retinas alters continuously due to the eyes’ involuntary microsaccades. The fixation’s small eye movements are essential in order to recalibrate the eye’s neuron sensors [135]. A qualitative evaluation of the eye tracking system used to record eye movements is shown in Figure 12e, which includes fixation time, count, and TTFF for each AOI [143]. As seen in Figure 12e, eye fixations and their duration frequently correspond with the respondent’s interest aspects in an image [143,149]. Therefore, by separating such components, quantitative analysis can produce data with a higher and lower ranking and order [143]. For more precise eye movement analysis, researchers attempted to compare different eye tracking metrics by quantitative fixation time and recognition [73]. In addition to the TFF method, first fixation duration (FFD), total fixation duration (TFD), and fixation count (FC) methods were used to analyze the detailed eye fixation. The FFD measures how quickly an object is recognized upon content identification. The shorter the period, the more effective information is transmitted. Total fixation counts and TFD are the metrics of time and count used to represent the participant’s distribution of interest in the target area [73]. The bigger the metrics TFD and FC indicate, the longer a participant focuses attention on the target object, and the more distribution of interest on the target across the entire scene [73]. The gaze and fixation points are more influenced by our own interests and experiences or by a user’s predetermined task. Visual scenes are perceived differently by different individuals. Early psychological research discovered a correlation between eye movements and visual attention [149,154]. The finding allows researchers to establish a foundation for measuring eye movements by observing the point of gaze, fixation, and saccades [143]. Some studies attempt to present visual information and continuous interpretation whenever the user opens their eyes and moves [72].

#### 3.2.2. Visualization and Analysis of Eye Movements

*Gaze Mapping and IR Technology*

**Figure 13:**
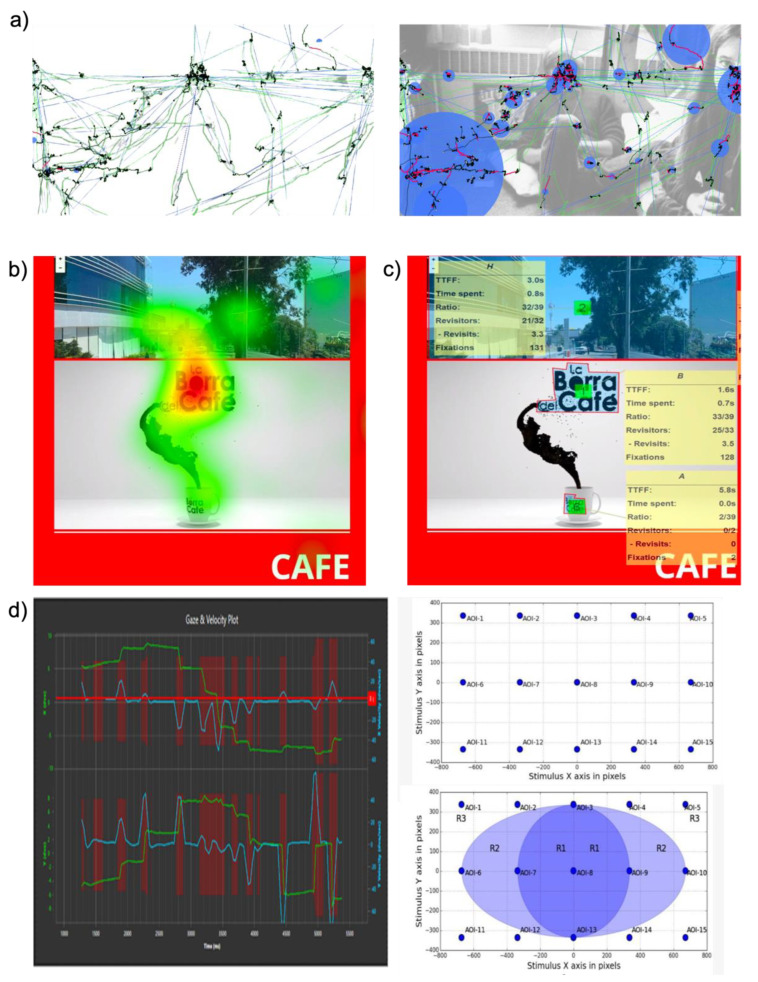
Eye tracking algorithms: (**a**) graphical overview of gaze tracking and mapping, overlapping data on user’s eye view (Reproduced under terms of the CC-BY license [167]. Copyright 2020, the Authors. Published by DOAJ). (**b**) Heatmap and dwell time analysis (Reprinted under terms of the CC-BY license [157]. Copyright 2022, the Authors. Published by MDPI). (**c**) Analyzed AOI from gaze and fixation data (Reprinted under terms of the CC-BY license [157]. Copyright 2022, the Authors. Published by MDPI). (**d**) Area of interest (AOI) model computed from ensembled eye tracking gaze, velocity, and fixation data (Reprinted under terms of the CC-BY license [166]. Copyright 2018, the Authors. Published by MDPI).

The gaze mapping uses time series plot maps, which show the sequential, step-by-step process of users’ visual search techniques [72]. A sequence of uniformly sampled, raw gaze points is transformed into a series of duration saccades and fixations using the eye gaze and mapping application [158]. Continuous fixations are detected by examining sequences of gaze point measurements that remain relatively consistent [72]. If a new gaze point lies within a circular region, a fixation is extended to include a new gaze point by running the average of an ongoing fixation [72,159]. Gaze plot maps can be generated using eye tracking systems such as the Tobii (Tobii AB, Stockholm, Sweden) eye tracking systems [160]. As shown in Figure 13a, horizontal and vertical gaze plot maps were generated by detecting microsaccades. The figure shows the individual ongoing fixation on the gaze point and saccades point with traces. The system can represent fixation locations as proportional circles, colored according to time, and the sequence of saccades between fixations as line symbols [158]. A gaze plot map shows the eye movements of a single user for a single image trial, thus providing a graphic overview of each user’s visual search strategy [158]. As shown in Figure 13a, microsaccade movements can be detected with a trace line [135]. A saccade is the fast movement of the eye. Saccades serve as a mechanism for rapid eye movement and fixation. They most frequently shift from 1-degree to 40-degree visual angles and last 30 ms to 120 ms. Between each saccade, there is typically a 100 ms to 200 ms delay [148]. The point light sources that illuminate the eye are modeled as having omnidirectional radiation. IR light-emitting sources are the primary light sources. Each light source consists of an array that corresponds to a single-point light source at the array’s center. The direction and position of the light sources are defined in comparison to the global coordinate system due to them being modeled as point light sources [158]. To define the gaze direction vector in the global coordinate system and to integrate it with the characteristics of the scene’s objects, the point of gaze (POG) is computed as the intersection of the vector with the screen. A mathematical model is used to calculate the corneal curvature’s center using the concepts of refraction and reflection [135,136,158]. Studies show that effective ways for detecting the POG could be approximated by using statistical averages for all eye characteristics in a single camera and a single light source [131,135,150]. Both spherical surface and non-spherical cornea models are used to obtain gaze estimations and to personalize eye parameters from the surface of the cornea model [135,150].

*Heatmaps*

A heatmap is a type of visualization technique that displays the variation of gaze points. Compared with a fixation map, a heatmap is a simple approach for quickly discovering what in the image is most interesting or where is more attractive than others [161]. A fixation heat map, as seen in Figure 13b, presents an overview of a compound image, including fixation locations and times [158]. Fixation heat maps and heat maps in general are influenced by cartographic traditions such as isoline and surface mapping [136,158]. By using gaze plots and heat maps, the obtained gaze fixation data are then viewed and evaluated [162]. Most cognitive activity occurs during fixations and not saccades, although some components of the visual scene are perceptually processed during saccades [158]. Studies that employ eye tracking analyses frequently concentrate on the data from heat maps. Commercial tools such as Tobii software create fixation heat maps by using red for areas in the image [158,161]. The software continuously acquires where users were fixated for a short period as green color in the image [161]. A fixation heat map provides a composite graphic showing the locations and lengths of fixations, and the variations in color value indicate the intensity of the time period [158]. Heat maps are also quantified by the center point for easy custom applications. To obtain a general idea of what is qualified, the user often estimates the length and width of the entire object before estimating the distance from a spot on the object to the dimension [158,161,162]. This analysis allows visual and statistical approaches for attention mapping and spatiotemporal eye tracking.

*Area of Interest (AOI) and Dwell Time*

In many eye tracking studies, researchers aim to classify and analyze how a user looks at a specific part of a stimulus, such as an object in a scene, a particular word in a sentence, or another human being [140,142,161]. To fulfill this goal, researchers identify an area of interest that corresponds to a region of interest. The target AOI is used to pick out particular parts of a visual stimulus and extract metrics for those regions. Figure 13c shows specific AOI spots and summary statistics such as eye fixation, duration, and repeating. Recent eye tracking metric attempts to connect and pattern numerous AOI information to identify user’s preferences and ranks inside the image [143]. The AOI spend time, and the statistical data of TTFF reflect what is more interesting but needs further analysis because it is not related to whether the assessment of the AOI is negative or positive [143]. Fixation counts, on the other hand, are positively correlated, implying that people tend to pay more attention to the image’s more appealing aspects [143]. Researchers proposed multiple eye tracking systems that reflect an interactive environment of visualizing analysis [163,164] or analyzing eye-movement protocols and object findings [165,166]. Recent eye tracking software is able to process complex eye movement variables, generate personalized eye interactions with objects, and analyze detailed areas of interest [142]. Figure 13d shows the eye tracking analysis steps from eye movement variables (user’s gaze plot, velocity plot, and fixation plot) to an AOI model. On the rectangular stimulus grid, the model indicates multiple locations of the stimulus map with colored areas. The AOI model shows a 2D Gaussian distribution of fixation and shadow mapping. This model map provides the meaning of eye movement by projecting object overlay and defining semantic localizations on the viewer’s target areas [165,166]. This real-time tracking system provides data alignment and classification between physiological information and eye tracking information.

The amount of time a user spends viewing an AOI is known as the dwell time. Researchers typically determine the average dwell duration, which informs how long a user spends on average viewing an AOI [73,133]. The length of dwell time depends on the size and informational density of the AOI. The complexity of the user’s scene and situational awareness also impacts dwell time. The dwell time is affected by the movement properties of the stimulus [168]. Related studies on eye movements indicate that bottom-to-top or top-to-bottom types of attention are strongly integrated [156,168,169]. If bottom-to-top and top-to-bottom influences on attention are independent of one another, then a simultaneous view might be anticipated [168]. It would be reasonable to assume that the eye’s attention moves to position A on some trials and to location B on others if top-to-bottom attention intends to guide the eyes to location A and bottom-to-up attention is drawn to location B. However, the previous results show that the eyes often move to a position between A and B in a similar situation [169]. As a result, the dwell time becomes a significant statistic because it can reveal information about the cognitive eye movements and intentions of a user [156].

### 3.3. Eye Tracking Platforms

Studies using eye tracking systems have grown significantly in both quantity and variety over the past decade [170]. There has been many prior research studies that attempt to track user and interpret intentional eye movement [170]. The early phase of eye tracking research laid on utilizing prior observations on eye movement, perception, seeing, and looking [74]. A new advancement in optical device-based mobile eye=tracking systems presents the comprehensive tracking of nonintrusive human gaze points [73,74]. Recent developments in real-time computer devices have led to the emergence of mobile and stationary eye tracker platforms and these platforms changed daily lives.

#### 3.3.1. Screen-Based

**Figure 14:**
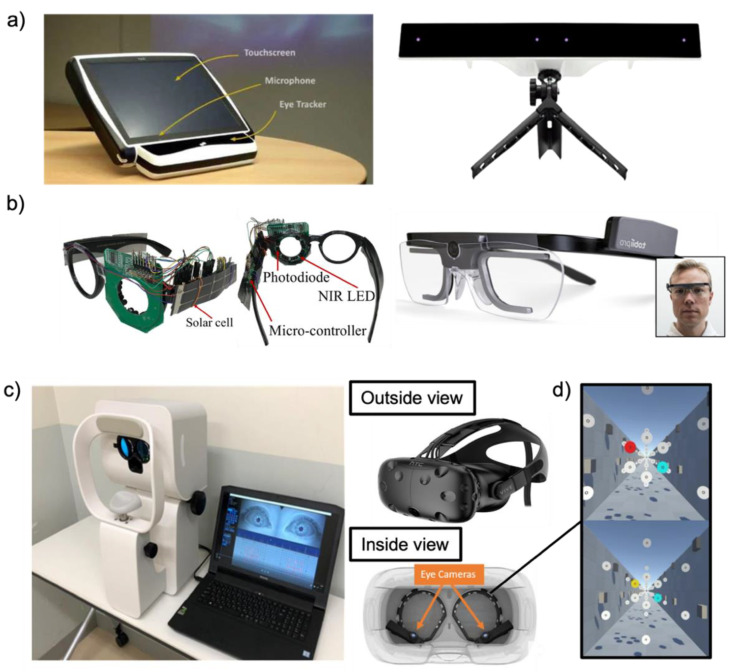
Eye tracking platforms. (**a**) Glass-type eye tracker (Left: Reprinted with permission [184]. Copyright 2013, Springer-Verlag London, Right: Reprinted under terms of the CC-BY license [3]. Copyright 2020, the authors. Published by MDPI). (**b**) Screen-based eye tracker (Left: Reprinted with permission [174]. Copyright 2022 ACM, Inc., Right: Reprinted under terms of the CC-BY license [160]. Copyright 2020, the Authors. Published by Springer Nature). (**c**) VR hardware platform (Left: Reprinted under terms of the CC-BY license [182]. Copyright 2020, the Authors. Published by DOAJ., Upper right: Reproduced under terms of the CC-BY license [3]. Copyright 2020, the Authors. Published by MDPI, Lower right: Reproduced under terms of the CC-BY license [183]. Copyright 2020, the Authors. Published by DOAJ). (**d**) Perceptual depth change with eye trackers in virtual reality (Reprinted under terms of the CC-BY license [177]. Copyright 2021, the authors. Published by Frontiers).

The majority of contemporary eye gaze-tracking devices track eye movement by processing visual information of the eyes digitally [135]. To track the POG, high-resolution eye images are necessary so that screen-based data acquisition system can be emerged [135,168]. In screen-based systems, infrared light is used to illuminate the eye, and produce glints for gaze direction estimation [74]. Moreover, the system analyzes the data of distance and experimental setups [168]. This methodology can be used for a wide range of evaluation methods that include measuring rotation, translation, pupil’s shape, location of the limbus, and corneal reflections by IR sources [135]. After the calibration of distance and light, the eye tracker data usually include the gaze position and conversion of screen coordination. The spatial accuracy of the eye is dependent on a range of motion in remote eye tracker platforms that use cameras to identify and track the eyes’ features [67]. In more recent systems, an estimated inaccuracy rate is approximately 1 degree or under in computing the optical target [67,135]. The POG for the projected scene that the user is viewing, is first calculated by the eye tracker using inputs from the scene. A correct understanding of the POG is the second prerequisite [135]. IR light sources are frequently functional elements in produced glints on the cornea that many commercial devices compute to track [154,160,170]. As seen in Figure 14a, a remote eye-gaze-tracking system consists of a CPU for data collections, an image camera, infrared light sources, and a screen for determining the subject’s eye focus, without a wired connection. The field of computer vision has long been active in the study of screen-based real-time eye recognition and tracking. The market currently offers a wide variety of gaze tracking hardware and software [171]. Recent research attempted to use an eye tracking system as part of PC accessories such as replacing mouse controllers. However, the eye tracking system has encountered limitations due to the placement of the camera or other devices near the screen [72,169,171].

#### 3.3.2. Glasses Type

Eye tracking glasses are infrared sensors and camera-integrated portable devices that can be easy to wear [172], as shown in Figure 14b. This unique platform allows the user to move freely with the head unit’s discretion and freedom for natural head movement [160]. The system utilizes an approach of light reflection from the pupil and captures eye image using cameras. Then, extensive image processing is used to determine the position of the pupil [168] in eye trackers with high performances [74]. Wearable eye trackers can record the user’s vision as well as their surroundings and background noise [74]. Compared to screen-based systems, wearable eye trackers have advantages in recording a person’s gaze movement in 3D real view [168,172]. With these advantages, mass data studies can be presented by large datasets for new clinical findings and interactions [173]. Existing commercialized wearable glasses such as Tobii and SMI (Imotions Inc, Boston, USA) made previous research on wearable eye trackers daily human circumstances possible [171,174]. Recent experiments used glass-tupe eye trackers, enable recording eye movements in natural settings when humans are moving freely [160]. This platform makes conducting numerous studies that are not appropriate for screen-based eye trackers possible, such as detecting eye contractions [175], tracking 3D gaze behavior to obtain coordinate information [162], attempting to develop a battery-free tracker for ubiquitous computing platforms [174], and evaluating human and robot interactions with active behavior [176]. Eye tracking has been used in numerous specialized software programs that are developed for various study fields.

#### 3.3.3. Virtual Reality (VR)

The usage of VR technology is growing across a range of applications, including immersive training, as well as in various fundamental research areas, such as cognitive science, visual perception, and psychology [177,178]. Eye tracking in VR analyzes multiple computations, including perceptual depth changes, vergence, and inter-pupillary changes [177,179]. In addition, distance virtual reality has the feature of a pre-calculated experiment setup allowing the subject to move freely in natural settings [179]. Thus, the VR platform can effectively integrate free eye movements and eye tracking methodology that suggests human-centered computing approach. Eye tracker in VR technology has been used to measure vergence eye movements and depth analysis [180,181]. Vergence, which is the simultaneous rotation of the eyes while viewing objects, is necessary for distance measurement, because of the perceived depth from monocular and binocular depth cues [179]. This depth perception requires fast and precise eye movements along with saccadic and fixation information [136,177]. Recent researchers used the vergence movement in response to depth changes combined with an eye tracker platform for precise clinical gaze direction studies, as demonstrated in Figure 14c [182]. In addition, VR’s optical information is more reliable than the glass type because of the unlimited geometries. It allows for the analysis of individuals’ behaviors with respect to the objects they looked at as well as the locations they looked at in relation to the behaviors they performed in the virtual environment [183]. With the technology of using gaze-detection technology, the device can measure gaze directions of nine in both eyes at the same time in VR eye platform [182]. This offers a dichoptic separation structure and allows the eye to integrate with specially designed screens, such as virtual reality environments. IPD, or the distance between the centers of the left and right eye pupils [179], is another crucial aspect of the human binocular visual system that changes with the object’s depth. By changing the IPD, this contour-based eye tracking data can distinguish between a verged and gaze distance from an object. The IPD value will become smaller if the eyes are focused at a close distance and it becomes larger if they are focused at long distances [179]. The IPD can track changes in perceptual depth in VR because it was recorded independently as eye tracker camera data [179]. By examining behavior, perception, and interest, an eye tracking integrated VR platform offers a chance to understand the human visual system [183]. One study that used an eye tracker in virtual reality to estimate perceived depth change is shown in Figure 14d. The top convergence case shows how the user’s eyes move their sight from bottom-to-top and top-to-bottom. This demonstrates how effective the platform’s angle computation is for measuring angles in 3D maps. With eye tracking and VR, it is feasible to compute a subject’s gaze in 3D space and to see where they are focusing while they are engaged in an activity. Unlike the real world where it is difficult to identify the regions of interest in 3D spaces and reconstruct the points when the regions were looked at, it is simple to do so in VR eye tracking [182]. It is possible that in the future, virtual and augmented reality glasses will widely use this 3D integration to simulate more realistic information [161].

### 3.4. Applications

Humans look at objects to receive visual information, which is then used to recognize events and objects to understand the situation [185]. Eye tracking is employed throughout many fields of psychology, medical examination, and cognitive science to study topics including oculomotor system development, attention, perception, disease diagnosis, diverse usability, and neurological findings [186]. Based on the different characteristics, this chapter introduces examples of cognitive-based medical and educational committing creative tasks by reading human intentions and assisting humans directly. Researchers can determine how effectively a person executes a task by examining attentional eye behaviors when communicating and working on a task. Additionally, observing the gaze allows estimating the individual’s cognitive states and understanding the user’s status [185]. These approaches play a significant role in an expanding number of applications [171]. Recent technological advancements with precise quantification, large data, and automated evaluation enable eye tracking applications available for disease diagnosis and assisting roles [70,133,153,154,171,187]. Multi-disciplinary research, including driving applications [134], pattern analysis with machine learning [71], human–computer interaction [67], and learning assistant and evaluation [151,178] are some examples of innovations.

#### 3.4.1. Cognitive Behavior and Human Recognition

**Figure 15:**
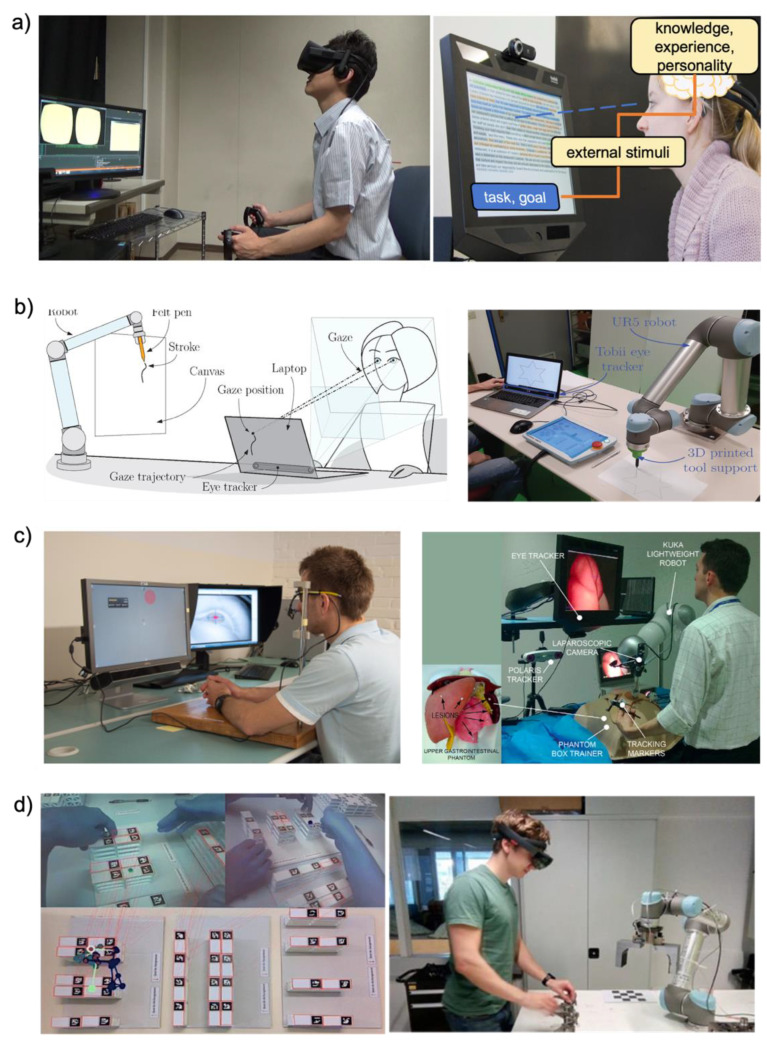
Examples of eye tracker applications. (**a**) Applications in cognitive learning and human recognition (Left: reprinted under terms of the CC-BY license [194]. Copyright 2020, the authors. Published by MDPI, Right: reprinted with permission [184]. Copyright 2013, Springer-Verlag London). (**b**) Robot operation with eye tracking (Reproduced under terms of the CC-BY license [193]. Copyright 2021, the authors. Published by MDPI). (**c**) Eye tracker application in operative simulation and surgical operation (Left: reprinted with permission [195]. Copyright 2012, Springer-Verlag Berlin Heidelberg, Right: reprinted under terms of the CC-BY license [13]. Copyright 1969, the authors. Published by Elsevier Ltd.). (**d**) Interface for task-related eye tracker application (Left: reprinted with permission [196]. Copyright 2018, International Society of the Learning Sciences, Inc., Right: reproduced under terms of the CC-BY-NC-ND license [197]. Copyright 2019, the authors. Published by Elsevier Ltd.).

Cognitive behavior can be revealed in a variety of ways, such as changes in eye movement, action, the inability to recognize people and objects, and even the loss of memory [170,185]. Researchers attempt to identify cognitive strategies (e.g., problem-solving) and recognition skills when humans execute tasks to quantify and qualify human recognition [185,188]. Recent researchers presented a tangible medium of diverse applications with eye movement analysis. Referring to Table 4, researchers attempt to measure gaze movements [182], artifacts [186], and wayfinding [189] to assess the user’s intention and real-time eye movement analysis. Additionally, for precise patient diagnosis [133,153,190,191] and behavioral research [160,192], wearable and screen-based tracking devices guide how deep investigations on human gaze behavior in real-world scenarios. The “individual difference” has gained significant attention in the fields of target content and psychology in recent years. Researchers emphasized the study of cognition and recognition, which is influenced by cognitive psychology, in order to identify personal behavior in a diverse medium [188]. The recent study analyzed the eye movement data of student participants with different individual cognitive styles when they read and recognized contents, as shown in Figure 15a. The study explores the differences in visual attention among individuals with different cognitive behaviors by identifying unique eye movements during interaction and communication [188]. This non-invasive eye tracking platform allows the easy coordination of test designs and stimuli provision, making it possible to collect robust eye information data [171]. While eye movements were previously measured and recorded in the laboratory, an advanced eye tracking platform proposes the use of human statuses and cognitive monitoring. Precise data acquisition mechanisms such as disease status and monitoring of disease progression are well established [70,153,154].

#### 3.4.2. Contents

Using eye tracking technology, a computer can precisely track the motion of the user’s gaze on a screen in real-time. People with physical limitations can use it as a natural and simple interface between themselves and outside technology, giving them a potential means of communication [193]. Recent attempts have been made to combine an eye tracker with traditional input methods, including a mouse, keyboard, controller, and speech recognition. Figure 15b shows one example where a robotic arm operates as a standalone hand for any point in the workspace by simply moving the user’s eyes [193]. This eye tracker integrated system shows HMI compatibility by creating intelligent space with the support of AR and VR. A system architecture for using an eye tracking interface can create new artistic mediums and content with an industrial robot, especially those targeting amputees, those with movement disorders, or people who need an assistive device for creative drawing and painting [193]. The researcher also presented enhanced imagery techniques for virtual web mapping for re-producing eye movements and human strategies [158]. It opens up the prospect of duplicating a person’s visual search approach via picture and strategy enhancements. The individual map identification analysis was possible by using precise eye analyses and qualitative and quantitative analytical methodologies [158].

#### 3.4.3. Guided Operation

As a result of significant advancements in eye tracking technology in recent years, eye trackers are highlighted to assist human operational tasks and supportive devices [198]. Studies using wearable eye tracking technologies, such as those attached to light eyeglass frames, have been able to help novice surgeons throughout various laparoscopic procedures, as shown in Figure 15c [198]. Additionally, recent research has shown that analyzing gaze and velocity provides prospective surgical risk and can help assess tasks during simulated or real operative procedures. The eye tracker’s integration in surgery identifies the task’s complexity and measures the cognitive change of the user [195]. Research findings, including the current surgery study, have shown the sensitivity of gaze-based metrics as an assessment tool [195] and it demonstrates the possibility of assisting surgical education, surgical robot, and operation assessment in order to produce a more effective and efficient health care system [195]. As shown in Figure 15d, the researcher attempts to construct a tangible interface for task-related eye tracker applications [197,198]. The researcher shows remote construction tasks with mobile eye trackers. The user can assist by remote eye information input. Interface proposes expandability on collaborative tasks, complex processes, and design interventions for safe and remote operation tasks [195]. Research in driver’s assistant technology utilizes eye gaze and fixation patterns to anticipate driver’s future maneuvers [134]. Real-time intention tracking enables smart and collaborative advanced driver assistance systems (ADAS) that can aid drivers to overcome safety critical situations [134]. Recently, researchers presented the tangible medium of diverse applications. Referring to Table 4, researchers attempt to guide operations with eye tracking with respect to surgical skill and training [13] and also with respect to driver guidance systems during driving operations [158]. Applications for supportive guidance provide educational breakthrough opportunities [151,198] and attention evaluation tools for learning purposes [134].

**Table 4:** Application examples using eye tracking systems.

Application		Target User	Platform	Device Info.	Gaze Detection	Processing Method	Refs.
Cognitive Recognition	Autism	Infant	Screen-based	ISCAN, Inc.	-Dark pupil Tracking -Corneal reflection	Customized (Eye position and fixation data identification with MATLAB)	[153]
	Impact of slippage	Any mobile user	Eyeglasses	-Tobii -SMI -Pupil-labs	-PCCR -Dark pupil Tracking -Corneal reflection	Commercial (Tobii Pro: Process with Two cameras and Six glints per eye, iViewETG: Three makers tracking from SMI) Customized (EyeRecToo: Open-source pupil Grip algorithm)	[160]
	The Effects of Mobile Phone Use on Gaze Behavior in Stair Climbing	Any mobile user	Eyeglasses	Tobii Glasses 2.0	-PCCR -Corneal reflection	Customized (Frame by frame image classification with MATLAB)	[192]
	Diagnosis and Measurement of Strabismus	Children	Screen-based	EyeTracker 4C	-PCCR -Corneal reflection	Customized (EyeSwift: IR and Image Processing)	[190]
	Measurement of nine gaze directions	Patient with strabismus	Screen-based	OMD	-Pupil and corneal reflection	Customized (Hess screen test)	[182]
	ADHD	ADHD Patient	Screen-based	Eyelink 1000	-Dark Pupil Tracking -Corneal Reflection	Customized (MOXO-dCPT Stimuli, AOI and relative gaze analysis)	[133]
	ADHD	ADHD Patient	Screen-based	TX300	-PCCR	Customized (Logistic regression Classification model for pupil analysis)	[191]
	Measurement of pupil size artefact (PSA)	Any mobile user	Screen-based	EyeLink 1000 Plus, Tobii Pro Spectrum (Glasses 2)	-Dark Pupil Tracking, -Corneal Reflection	Commercial (Tobii Pro: Process with Two cameras and Six glints per eye)	[186]
	A comparison study of EXITs design in a wayfinding system	Any mobile user	Eyeglasses	Tobii Glasses	-PCCR	Customized (Custom IR Marker, AOI Analysis)	[73]
Contents Creation	Artistic Drawing	Graphic user include people with diabilities	Screen-based	Tobii Eye Tracker 4C	-PCCR	Commercial (Tobii Pro: Process with Two cameras and Six glints per eye)	[193]
	To Enhance Imagery Base maps	Map User	Screen-based	Tobii Pro Spectrum	-PCCR	Commercial (Tobii pro: Process with Two cameras and Six glints per eye) Customized (AOI statistic and heatmap)	[158]
Guided operation supportive guidance	Semi-Autonomous Vehicles	Driver	Screen-based	faceLAB	-Pupil Tracking	Customized (Markov model, Pattern analysis)	[134]
	To capture joint visual attention	Co-located collaborative learning groups	Eyeglasses	SMI ETG	-Pupil/CR -Dark pupil tracking	Commercial (Fiducial tracking engine)	[196]
	Human robot interaction for laparoscopic surgery	surgeon	Screen-based	Tobii 1750	-PCCR	Customized (Hidden Markov model)	[13]
	Surgical Skills Assessment and Training in Urology	surgeon	Eyeglasses + VR	Tobii Glasses 2.0	-PCCR	Commercial (UroMentor simulator)	[198]
	Architectural Education	Ordinary Users, Students and Lecturers	Eyeglasses Screen-based VR	-Tobii -SMI	- PCCR - pupil/CR, dark pupil tracking	Commercial (BeGaze software)	[151]
	Education	Student	VR	Self-made “VR eye tracker”	-Record the condition of the pupils via infrared LED light	Customized (Analysis of regions of interest)	[188]

## 4. Discussion

We summarized device technologies and HMI applications in eye tracking. Previous EOG systems were bulky and used many wires with Ag/AgCl gel electrodes. The electrode can record signals in high fidelity, but it has issues, including poor breathability, skin irritation, and a loss of performance during long-term usage. Moreover, EOG signals have difficulties when detecting the detailed modality of input signals, so there is a limit to classifying various eye angles and eyes. Due to these limited capabilities, the HMI shown in previous studies only applies to simple motion control with a finite number of directions. Screen-based eye trackers were also used for HMI, but they required complex eye movements that caused extreme eye fatigue. Recent advances in electrophysiological signal monitoring and manufacturing of wearable platforms and various types of electrodes have enabled EOG monitoring systems with comfortable wearable EOG devices to detect eye movements without skin issues. Many research groups have introduced biocompatible electrodes to solve the skin issue of conventional gel electrodes. Various biocompatible electrodes were introduced, such as hydrogel, fiber, polymer, and micro-patterned types. These biocompatible electrodes enable long-term EOG measurements and multiple types of wearable platforms, such as glasses, face masks, headbands, and earplugs. Video monitoring systems have also been improved for eye tracking used in HMI applications. 

Based on recent technological advances, HMI applications via EOG show the potential for healthcare and virtual world development. We want to introduce two highly influential potential future usage. An instance of a healthcare application is the diagnosis of blepharospasm, which is the abnormal contraction of the eyelid muscles. Currently, there is no simple quantitative system for accurate and objective diagnosis of blepharospasm. To diagnose blepharospasm, EMG and EOG signals could be measured simultaneously with biocompatible electrodes. However, only using biopotentials is not easy to detect all clinical symptoms, such as the frequency of blinking, the duration of eye closures during spasms, and the combinations of blinking and spasms. In this case, the accurate diagnosis of blepharospasm will be possible via biopotentials with a camera-based eye tracker. Camera-based eye trackers can capture tiny eye movements that are difficult to catch via biopotentials. Moreover, biopotentials can measure hidden eye movements that the camera-based eye tracker cannot record. With this new mechanism, it is possible to increase the accuracy of the diagnosis of blepharospasm while supplementing each other’s limitations. 

Second is an integration of EOG technology and other biopotentials in the virtual world, such as a metaverse. The current virtual world platforms require complex user input to enjoy applications. For example, when moving the user’s location, users have to press and indicate the location where the user wants to go. For the interaction choice, both hands are the majority input source that requires clicking and moving hands. In order to expand the application capability of complex user inputs, biopotential signals can create new commands input. For people with disabilities who cannot move their muscles such as their hands or mouth, they can freely move in the virtual world with only EEG and EOG. Recent EEG technologies proposed reliable select mechanisms for long words and sentences. Through EEG technologies, the user can communicate with other users without speaking. In addition, EOG and EEG data in specific frequencies can track the user’s gaze and provides an additional command input mechanism that corresponds to the user’s additional behavior. Simple movements such as up, down, left, and right can be performed quickly by analyzing the EOG signal in the virtual world.

Eye tracking is in its early stage. Many studies and industries show the potential of ultimate HMI applications and next-generation diagnosis via recognition, sensing, and analysis. Researchers propose scrutinizing human intentions and integrating those intentions to actuate the task, suggest decision guidelines, and assist during operations. However, eye tracker technology is an insufficient data acquisition system for executing advanced and complex structures such as exoskeletons. Moreover, the limitations of eye tracking measurements using optical devices prevent it from becoming a primary parameter for clinical-level diagnoses. However, recent machine learning and advanced computing technology have shown the possibility of designing personalized profile modeling. Advanced technology makes the eye tracker suitable for various HMI applications, for new medical guidelines, or for understanding of a person’s cognitive state. Nevertheless, many aspects of eye tracking must be further developed to realize its applications in everyday life in terms of eye tracking usability and opportunities. Moreover, EOG data and eye tracker gaze data can be integrated with machine learning or each other for scalability and performance. We believe that the consideration of these challenges will provide broad scalability to further develop eye tracking for practical applications.

## 5. Conclusions

This paper summarizes various wearable EOG devices and eye-tracking systems in terms of material properties, sensing performances, and platform technologies. Specifically, we outline recent developments in biocompatible materials, manufacturing technologies, signal-processing strategies, integrated systems, and applications in detecting eye movements. Advances in wearable technologies and video monitoring systems for electrophysiological signal monitoring enabled various human–machine interfaces. The unique properties of flexible soft electrodes offer enhanced skin compatibility, long-term stability, and increased skin–electrode contact. Overall, soft material-enabled electronics and camera-based high-resolution systems are up-and-coming tools for accurately detecting eye movements and persistent human–machine interfaces.

## Figures and Tables

**Table 3:** Summary of applications using EOG signals.

Purpose	Target User	Signal	Data Processing	Refs
Wheelchairs	Disabled people	EOG + EEG + EMG	Signal processing	[51]
		EOG	LDA	[1]
		EOG	Signal processing	[4]
		EOG + EEG + EMG	Signal processing	[52]
Game controller	Anyone	EOG	DWT	[5]
		EOG	SWT	[60]
		EOG + EEG + EMG	SVM	[47]
		EOG	Signal processing	[61]
Drone		EOG	Signal processing	[11,59]
Virtual keyboard		EOG	SVM	[38]
		EOG+EEG+EMG	Signal processing	[51]
		EOG+EEG	SVM	[34]
		EOG	Signal processing	[62]
ADHD	Children	EOG	Signal processing	[64]
		EOG	Signal processing	[65]
		EOG	WT	[66]
Emotion Recognition	Anyone	EOG	SVM	[127]
		EOG + EMG	SVM	[128]
		EOG + Eye image	STFT	[126]
sleepiness	Driver	EOG+EEG	GAN + LSTM	[63]
Drowsiness	Anyone	EOG	Signal processing	[55]
		EOG+EEG	Signal processing	[58]
Sleep monitoring		EOG+EEG+EMG	Signal processing	[41]
		EOG	Linear classifier	[40]

## Data Availability

Not applicable.

## Abbreviation

ADHD	attention deficit hyperactivity disorder
AJP	aerosol jet printing
AOI	area of Interest
BCI	brain–computer interface
CEFs	conductive elastomeric filaments
CNN	convolution neural network
CNTs	carbon nanotubes
CVD	chemical vapor deposition
DI	deionized
DWT	discrete wavelet transform
ECG	electrocardiogram
EEG	electroencephalogram
EMG	electromyography
EOG	electrooculograms
EPEDs	epidermal paper-based electronic devices
FC	fixation count
FFD	first fixation duration
GO	graphene oxide
HCI	human–computer interaction
HMI	human–machine interface
HPC	hydroxypropyl cellulose
IPD	inter-pupillary distance
IR	infrared
LDA	linear discriminant analysis
MR	mixed reality
PCBs	printed circuit boards
PCCR	pupil center-corneal reflection
PDMS	polydimethylsiloxane
PMMA	poly methyl methacrylate
POG	point of gaze
PVA	polyvinyl alcohol
rGO	reduced graphene oxide
SNR	signal-to-noise ratio
SVM	supporting vector machines
TFD	total fixation duration
TTFF	time to first fixation
UV	ultraviolet
VR	virtual reality
